# Deep Learning Model to Classify and Monitor Idiopathic Scoliosis in Adolescents Using a Single Smartphone Photograph

**DOI:** 10.1001/jamanetworkopen.2023.30617

**Published:** 2023-08-23

**Authors:** Teng Zhang, Chuang Zhu, Yongkang Zhao, Moxin Zhao, Zhihao Wang, Ruoning Song, Nan Meng, Alisha Sial, Ashish Diwan, Jun Liu, Jason P. Y. Cheung

**Affiliations:** 1 Digital Health Laboratory, School of Clinical Medicine, Faculty of Medicine, The University of Hong Kong, Hong Kong Special Administrative Region, China; 2 School of Artificial Intelligence, Beijing University of Posts and Telecommunications, Beijing, China; 3 SpineLabs, St George and Sutherland Clinical School, University of New South Wales, Sydney, Australia; 4 Department of Orthopaedics and Traumatology, The University of Hong Kong, Hong Kong, China; 5 Spine Service, Department of Orthopaedic Surgery, St George Hospital Campus, Sydney, Australia

## Abstract

**Importance:**

Adolescent idiopathic scoliosis (AIS) is the most common pediatric spinal disorder. Routine physical examinations by trained personnel are critical to diagnose severity and monitor curve progression in AIS. In the presence of concerning malformation, radiographs are necessary for diagnosis or follow-up, guiding further management, such as bracing correction for moderate malformation and spine surgery for severe malformation. If left unattended, progressive deterioration occurs in two-thirds of patients, leading to significant health concerns for growing children.

**Objective:**

To assess the ability of an open platform application (app) using a validated deep learning model to classify AIS severity and curve type, as well as identify progression.

**Design, Setting, and Participants:**

This diagnostic study was performed with data from radiographs and smartphone photographs of the backs of adolescent patients at spine clinics. The ScolioNets deep learning model was developed and validated in a prospective training cohort, then incorporated and tested in the AlignProCARE open platform app in 2022. Ground truths (GTs) included severity, curve type, and progression as manually annotated by 2 experienced spine specialists based on the radiographic examinations of the participants’ spines. The GTs and app results were blindly compared with another 2 spine surgeons’ assessments of unclothed back appearance. Data were analyzed from October 2022 to February 2023.

**Exposure:**

Acquisitions of unclothed back photographs using a mobile app.

**Main Outcomes and Measures:**

Outcomes of interest were classification of AIS severity and progression. Quantitative statistical analyses were performed to assess the performance of the deep learning model in classifying the deformity as well as in distinguishing progression during 6-month follow-up.

**Results:**

The training data set consisted of 1780 patients (1295 [72.8%] female; mean [SD] age, 14.3 [3.3] years), and the prospective testing data sets consisted of 378 patients (279 [73.8%] female; mean [SD] age, 14.3 [3.8] years) and 376 follow-ups (294 [78.2%] female; mean [SD] age, 15.6 [2.9] years). The model recommended follow-up with an area under receiver operating characteristic curve (AUC) of 0.839 (95% CI, 0.789-0.882) and considering surgery with an AUC of 0.902 (95% CI, 0.859-0.936), while showing good ability to distinguish among thoracic (AUC, 0.777 [95% CI, 0.745-0.808]), thoracolumbar or lumbar (AUC, 0.760 [95% CI, 0.727-0.791]), or mixed (AUC, 0.860 [95% CI, 0.834-0.887]) curve types. For follow-ups, the model distinguished participants with or without curve progression with an AUC of 0.757 (95% CI, 0.630-0.858). Compared with both surgeons, the model could recognize severities and curve types with a higher sensitivity (eg, sensitivity for recommending follow-up: model, 84.88% [95% CI, 75.54%-91.70%]; senior surgeon, 44.19%; junior surgeon, 62.79%) and negative predictive values (NPVs; eg, NPV for recommending follow-up: model, 89.22% [95% CI, 84.25%-93.70%]; senior surgeon, 71.76%; junior surgeon, 79.35%). For distinguishing curve progression, the sensitivity and NPV were comparable with the senior surgeons (sensitivity, 63.33% [95% CI, 43.86%-80.87%] vs 77.42%; NPV, 68.57% [95% CI, 56.78%-78.37%] vs 72.00%). The junior surgeon reported an inability to identify curve types and progression by observing the unclothed back alone.

**Conclusions:**

This diagnostic study of adolescent patients screened for AIS found that the deep learning app had the potential for out-of-hospital accessible and radiation-free management of children with scoliosis, with comparable performance as spine surgeons experienced in AIS management.

## Key Points

**Question:**

Can a validated deep learning model facilitate diagnosis and management of adolescent idiopathic scoliosis (AIS) without extra radiation exposure?

**Findings:**

This diagnostic study including data from 2158 patients found that the deep learning–powered model had similar or improved sensitivity and negative prediction values in evaluating AIS severity and progression risk compared with spine surgeons.

**Meaning:**

These findings suggest that the deep learning model used in this study could assist in monitoring AIS, with potential to avoid delayed treatment and additional radiation exposure in growing children.

## Teaser

This diagnostic study assesses the ability of an open platform application using a validated deep learning model to classify and monitor idiopathic scoliosis in adolescents using a smartphone photograph.

## Introduction

Adolescent idiopathic scoliosis (AIS) manifests as a 3-dimensional spinal malformation.^1^ It is reported to occur in up to 2.2% of boys and 4.8% of girls,^2^ with a high prevalence of progression during puberty leading to reduced quality of life and mobility in adulthood, as well as cardiopulmonary impairment and back pain. Thus, early detection, close follow-up, and proper interventions are critical. Hong Kong has initiated a school screening program^3^ beginning in 1995 as a part of the territory-wide annual comprehensive health assessment scheme for AIS. However, this type of screening practice can be interrupted (such as during the COVID-19 pandemic), which underpins an increased need for out-of-hospital, accessible assessment.^4^

Furthermore, current AIS detection and follow-up require extensive clinical expertise. Available assessment tools include physical examinations and radiographs.^5^ Physical examinations assess shoulder height, waist asymmetry, thoracic cavity asymmetry, and rib and breast deformity. However, these assessments of the external appearance are subjective and can not reliably detect the specific malformation severity and type. Thus, further radiographic examinations are necessary^6^; the Cobb angle can be calculated automatically using deep learning on biplanar stereoradiography, offering consistent results with reduced radioexposure.^7,8,9,10^ Moreover, repeated radiographic examinations are required for monitoring AIS progression, which may carry unwanted consequences of increased radioexposure.^11,12,13,14,15^

Conventional radiation-free approaches in detection include Moiré^16^ topography (with arguable accuracy) and standardized photographs of the patient’s back. A study using images acquired via professional cameras in a controlled environment with clear background^17^ lacked accessibility as well as prospective validations. These limitations are largely due to the lack of paired data and a focus on the process and method rather than evaluation as a disease monitoring tool. Automated detection and classification of AIS using easily accessible smartphone images of patients is an option for out-of-hospital assessment but is challenging due to several factors. First, smartphone images introduce variability, including vibration, angle, and noisy background, making classification challenging. Second, the back of an individual with spinal malformation has variable appearances subject to different severity and curve types. We overcame this challenge by developing a virtual spinal evaluation platform (eFigure 1 in Supplement 1) called *AlignProCARE*,^18^ as there was a need for out-of-hospital spine malformation evaluation during the COVID-19 pandemic.^19^ By using the criterion standard disease severity taxonomy, ie, pathologies classified on real radiographs as ground truths (GTs) and a validated deep neural network (ScolioNets) model trained and validated internally, the AlignProCARE application (app) accepts arbitrary scenes, and it is trained end-to-end directly from GT labels and images for automated and mobile scoliosis classification with no extra radiation. Our platform has the potential to assist identification of scoliosis severity and therefore reduce the chance of radiographic screening for patients without scoliosis or with mild scoliosis.

The primary objective of this study was to demonstrate the deep learning model’s reliability using prospectively collected data from patients with AIS treated at another clinic. Additional clinical utility in distinguishing malformation progressions were evaluated as well.

## Methods

For this diagnostic study, the recruitment, use, analyses, and prospective testing of the radiographic images were approved by the University of Hong Kong institutional ethics committee. All participants provided written informed consent. This study is reported followed the Checklist for Artificial Intelligence in Medical Imaging (CLAIM) reporting guidelines.

### Data Collection

**Figure 1.**
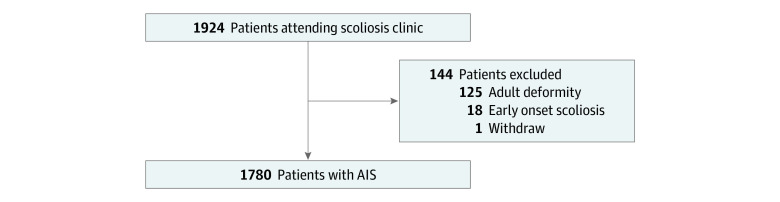
Participant Recruitment Flowchart

Participants were excluded if they had any prediagnosed systematic neural disorders that might influence their mobility (eg, prior cerebrovascular accident, Parkinson disease, myopathy). Other exclusion criteria were diagnosed with or having any signs of psychological disorders that might influence the adherence with the study, having any oncological diseases, having severe skin disorders or lesions on the back, having any other systematic diseases, being unable to complete the consent process, or having body mass index (BMI; calculated as weight in kilograms divided by height in meters squared) greater than 30. More detailed inclusion and exclusion criteria are reported in eAppendix 1 in Supplement 1. All participants meeting the inclusion and exclusion criteria were recruited with written consent prior to data collection (Figure 1), allowing for secondary analysis with all data anonymized and stored securely.

All images of participants’ backs were taken voluntarily by the participant’s parent or guardian using a smartphone. Minimal training was required to use the mobile app, as the app provided a built-in protocol for photo acquisition (eFigure 2 in Supplement 1). The paired radiographs were anonymously retrieved from the hospital system in PNG format, which were taken as a routine practice without extra experimental radiographs. Using the criterion standard severity classification,^20,21^ the ground truth (GT) labels of all data (cohort 1 for model development and cohort 2 for prospective testing) were provided by 2 spine specialists with more than 20 years’ experience in AIS management (A.D. and J.P.Y.C.) by manual annotation of the coronal radiographs.

The severity of AIS was defined by degrees, measured by the Cobb angle on coronal radiographs following the clinical criterion standard, which is the primary consideration for treatment planning.^21^ AIS severity is differentiated into 3 classes: no or mild AIS, defined as Cobb angle 20° or less; moderate AIS, Cobb angle 20° to 40°; and severe AIS, Cobb angle greater than 40°^20^ (eFigure 3 and eAppendix 2 in Supplement 1). The treatment planning recommendations were drawn based on the severity classifications. Curve types were subsequently decided from the GT labels by the location of the apical vertebra. Participants with a single curve (eFigure 3 in Supplement 1) were classified as having thoracic (T) curve or the thoracolumbar or lumbar (TL/L) curve.^22^ Patients with more than 1 curve were classified as mixed curve (eFigure 3 in Supplement 1). For follow-up assessments, the Cobb angle increment was checked during the follow-up examination to determine whether the curve was progressive or nonprogressive. A progressive curve is commonly defined as a curve magnitude increment increase of more than 5°, measured by the Cobb angle in 6-month follow-up intervals.^23^ Progressive curves require close monitoring and frequent follow-up visits. Especially during a growth spurt, curve progression is rapid and interventions need to be promptly introduced.^24^

To assess the ability of spine surgeons in distinguishing AIS clinical needs based on visual assessments without radiographic examinations, the severities and curve types of the prospective cohort 2 were blindly assessed by 2 additional spine surgeons using visual assessment of photographs of the participants’ backs. For participants with follow-ups, the back images from 2 visits were compared by the specialists to decide whether the curve was progressive. The senior surgeon has more than 20 years’ clinical experience with scoliosis, whereas the junior surgeon has less than 5 years’ clinical experience. The severity classification, curve types, and progressions were recorded independently for the blinded assessors, and their performances were compared with the GT labels obtained from radiographic examinations.

### Data Preprocessing and Model Development

The participants included in cohort 1 were recruited between October 2018 to September 2020 and used to develop the deep learning model for classifying scoliosis severity and curve types (eAppendix 3 in Supplement 1). Cohort 2 was recruited to prospectively test the performance of the model with 6 months of follow-up. We first used video matting method^25,26,27^ to achieve back segmentations on the images taken by smartphones, and then empirically cropped the segmented images to keep only the back with arms in the image to achieve improved classification performance.

Multilayer convolutional neural networks with attention mechanisms^28,29^ and multi-task strategies were developed and compared (eAppendix 4, eAppendix 5, eFigure 4, and eTable 1 in Supplement 1). In cohort 1, 1429 images were used for model training, and during training, we resized each image to 3 × 224 × 224 pixels to make it compatible with the original dimensions of the network architecture. We trained triple classification models directly for the severity classifications and binary classification models for the curve type classification (eFigure 5 in Supplement 1). Mixed curve type was classified if both curve types were present on 1 image. Receiver operating characteristic (ROC) curves were generated with each class as a positive class to calculate the areas under the ROC curves (AUCs) (eAppendix 6 in Supplement 1).

### Model Prospective Testing and Explicability

The prospective testing data set of 378 images (cohort 2) was not used during the model development or the in-house validation. No data augmentation or resampling was done to ensure a true validity of the prospective experiments in the testing phase.

To improve the interpretability of the model and mine the decision logic of the model, the class activation mapping (CAM) method^30^ was proposed, and some interpretable algorithms based on CAM^31,32^ were proposed afterwards. For the explicability of the model, we use the Score-CAM^33^ algorithm to explain the decision of the model. The difference between the probability of the masked image and that of the original image in the predicted label is used as the weight of the corresponding feature map. We normalized and up-sampled all the feature maps, and then linearly superimposed the feature maps according to their weights to get an interpretable heatmap.

### Statistical Analysis

To evaluate the performance of the model, true positive (TP), true negative (TN), false positive (FP), and false negative (FN) values were calculated.^26,27^ Evaluation metrics included sensitivity (calculated as TP / [TP + FN]), specificity (calculated as TN / [TN + FP]), positive predictive value (PPV; calculated as TP / [TP + FP]), negative predictive value (NPV; calculated as TN / [TN + FN]), and accuracy (calculated as [TP + TN] / [TP + TN + FP + FN]).

The ROC curve was plotted based on the final sensitivity scores and 1 – specificity scores with different thresholds, and the AUC was computed from the ROC curve as:
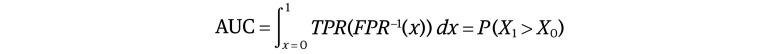
where *X_1_* is a positive instance and *X_0_* is a negative instance; *TPR* represents true positive rate and is equal to sensitivity; *FPR* represents false positive rate and equals 1 – specificity. The ROC curve visualizes TPR vs FPR in a graphic, and the metric AUC denotes the AUC with a range between 0.5 and 1. The closer the AUC is to 1.0, the higher the authenticity of the detection method; equal to 0.5, the lowest authenticity and has no application value.

To evaluate the agreement between groups of paired samples with unknown distribution, the Wilcoxon signed-rank test (2-sided hypothesis) was performed. We used the stats.wilcoxon function in SciPy software version 1.7.1 (SciPy) to assess the interrater agreement between the 2 spine surgeons and the model on using back appearance classifying the scoliosis severity and curve type. The same practice was performed to compare the agreements between different deep learning models (eTable 2 in Supplement 1). *P* < .0001 was considered statistically significant. We also used Python version 3.8 (Python Software Foundation), and NumPy version 1.22.4 (NumPy). Data were analyzed from October 2022 to February 2023.

## Results

### A Summary of Data Set

Between October 2018 and September 2020, 1780 participants (mean [SD] age, 14.3 [3.3] years; range 10-18 years; 1295 [72.8%] female; mean [SD] height 161.2 [9.1] cm; mean [SD] body weight, 48.4 [10.8] kg) of 1924 participants attending a tertiary referral center were recruited and eligible to populate cohort 1 (Table 1) for the development of the model. For the prospective testing cohort (cohort 2), 378 patients (mean [SD] age, 14.3 [3.8] years; range 10-18 years; 279 [73.8%] female; mean [SD] height, 159.1 [9.6] cm, mean [SD] body weight, 46.5 [9.8] kg) (Table 1) were recruited from participants consecutively attending AIS clinics from October 2020 to March 2022 and were assessed by the model. Among 2158 participants in both cohorts, 652 participants (30.2%; mean [SD] Cobb angle: 3.9° [1.2°]) required no intervention, 1250 participants (57.9%; mean [SD] Cobb angle: 16.1° [6.3°]) required nonsurgical interventions with regular follow-ups, and 256 participants (11.9%; mean [SD] Cobb angle: 48.7° [22.6°]) were under consideration for surgery. Additionally, participants completed a total of 376 follow-up visits (mean [SD] age, 15.6 [2.9] years; 294 [78.2%] female; mean [SD] height, 161.5 [8.9] cm; mean [SD] body weight, 47.3 [8.7] kg) during the study period. No patient had more than 1 follow-up visit.

**Table 1.** Data Characteristics of the Training and Prospective Testing Cohorts

Type or feature	Training cohort, No.		Validation cohort, No.		Follow-up visits, No.		Clinical implications
	Patients	Radiographs	Patients	Radiographs	Patients	Radiographs	
Curve severity							
No or mild (Cobb angle <20°)	555	1104	97	191	102	217	No intervention required. For the skeletally immature, regular follow-up is required every 4-6 mo to identify curve progression early, for which bracing may be recommended.
Moderate (Cobb angle 20°-40°)	1055	2109	195	390	237	526	Bracing required to prevent curve progression if still skeletally immature. No intervention is required at the end of growth. Scoliosis-specific exercises may also be prescribed.
Severe (Cobb angle >40°)	170	337	86	172	37	106	Risk of adulthood progression. Surgical intervention may be required in the form of vertebral body tethering (skeletally immature only) or curve correction and spinal fusion.
Curve type							
T (single curve)	118	374	38	76	30	59	More likely to develop chest wall deformities and unleveled shoulders
TL/L (single curve)	385	767	65	129	94	226	More likely to develop pelvic obliquity and waistline deformities
Mixed curve (>1 curves)	1153	2303	262	524	248	559	Often more balanced. Unequal sizes may lead to more deformities for T major curves or TL/L curves

Abbreviations: T, thoracic; TL/L thoracolumbar and lumbar.

All 2158 participants had radiographic and routine clinical assessment data with an extra photograph taken of their back (eFigure 3 in Supplement 1). From the original radiographic images in Digital Imaging and Communications in Medicine format, 4303 radiographic images in portable network graphics format were exported from the picture archiving and communication system of the hospital (eFigure 6 in Supplement 1), including 1295 images (30.1%) for participants who had no intervention, 2499 images (58.1%) for participants who had nonsurgical interventions, and 509 images (11.8%) for participants under surgical consideration. Among participants in cohort 1 classified by spine surgeons via radiographic images, 555 participants (31.2%) were classified as having no or mild AIS, 1055 participants (59.3%) were classified as having moderate AIS, and 170 participants (9.6%) were classified as having severe AIS. For cohort 2, 97 participants (25.7%) were classified as having no or mild AIS, 195 participants (51.6%) were classified as having moderate AIS, and 86 participants (22.7%) were classified as having severe AIS (eFigure 7 in Supplement 1).

Despite 54 eligible participants with no AIS and no curves in cohort 1, the remaining 1726 participants included 188 participants (10.9%) with T curves, 385 participants (22.3%) with TL/T curves, and 1153 participants (66.8%) with mixed curves. For cohort 2, excluding the 13 participants with no AIS and no curves, the remaining 365 participants included 38 participants (10.4%) with T curves, 65 participants (17.8%) with TL/T curves, and 262 participants (71.8%) with mixed curves. Different curve types demonstrated different appearance features discerned by spine surgeons, with the T curve consisting of rib humps, chest wall malformations, and unleveled shoulders, whereas the TL/L curves developed unbalanced pelvic and waistline malformations (Table 1; eFigure 3 in Supplement 1). The physical appearance features were not used in the model training, and the curve type GTs of all participants were obtained via radiographic assessment.

### Prediction Accuracy During Prospective Testing

**Figure 2.**
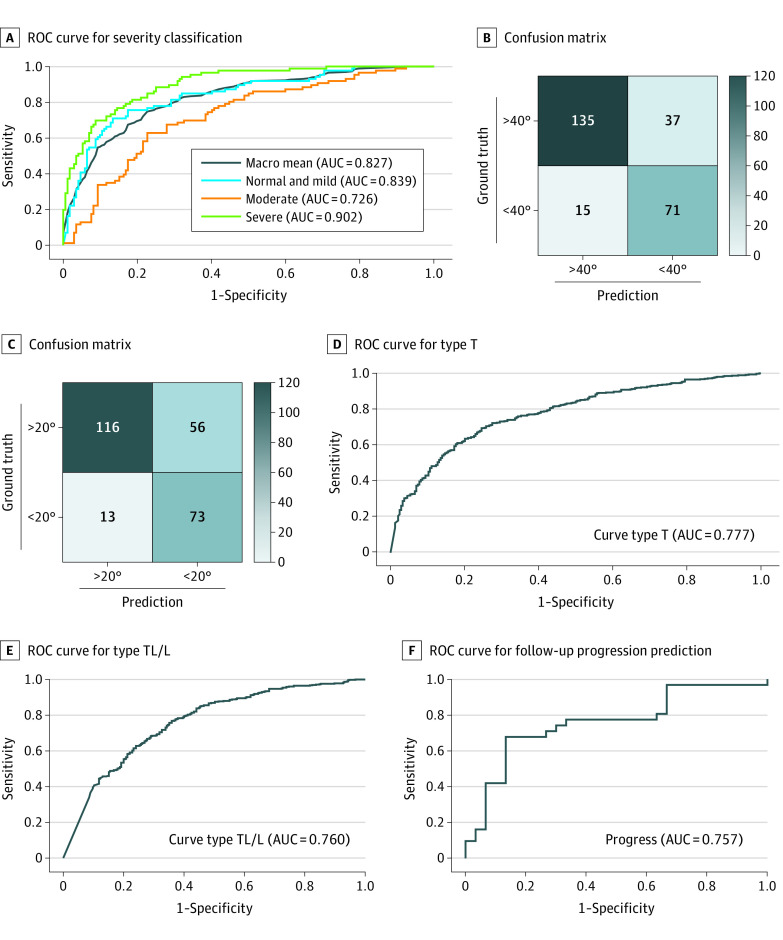
ROC Curve and Confusion Matrix for Prospective Test B, The confusion matrix corresponds to the final follow-up severity classification results. C, The confusion matrix corresponds to the final consider surgery severity classification results. AUC indicates area under the receiver operating characteristic (ROC) curve; T, thoracic; TL/L, thoracolumbar and lumbar.

In prospective testing, the model predicted no or mild AIS (ie, no interventions) with an AUC of 0.839 (95% CI, 0.789-0.882) and severe AIS (ie, considering surgery) with an AUC of 0.902 (95% CI, 0.859-0.936) (Figure 2A and Table 2; eTable 3 and eTable 4 in the Supplement). Confusion matrices were generated to visualize the agreement between actual and predicted results (Figure 2B). We found that the model correctly recognized AIS severities as well or better than the surgeons’ estimates (sensitivity for recommending follow-up: model, 84.88% [95% CI, 75.54%-91.70%]; senior surgeon, 44.19%; junior surgeon, 62.79%; sensitivity for recommending considering surgery: model, 82.56% [95% CI, 72.87%-89.90%]; senior surgeon, 20.93%; junior surgeon, 19.76%; NPV for recommending follow-up: model, 89.22% [95% CI, 84.25%-93.70%]; senior surgeon, 71.76%; junior surgeon, 79.35%; NPV for recommending considering surgery: model, 90.00% [95% CI, 84.95%-93.48%]; senior surgeon, 70.43%; junior surgeon, 70.51%) (Table 2; eTable 3 in Supplement 1).

**Table 2.** Performance Evaluation Metrics of ScolioNets and Surgeons on the Prospective Data Set in Distinguishing Curves Requiring No Interventions or Follow-Up Using Single Back Photographs

Method	%					AUC
	Sensitivity	NPV	Specificity	PPV (%)	Accuracy (%)	
ScolioNets, % (95% CI)	84.88 (75.54-91.70)	89.22 (84.25-93.70)	67.44 (59.89-74.38)	56.59 (50.81-62.20)	73.26 (67.41-78.56)	0.839 (0.789-0.882)
Senior surgeon	44.19	71.76	70.93	43.18	62.02	NA
Junior surgeon	62.79	79.35	71.51	52.43	68.60	NA

Abbreviations: AUC, area under the receiver operating characteristic curve; NA, not applicable; NPV, negative predictive value; PPV, positive predictive value.

The model achieved a curve type prediction AUC of 0.777 (95% CI, 0.745-0.808) for T, 0.760 (95% CI, 0.727-0.791) for TL/L, and 0.860 (95% CI, 0.834-0.887) for mixed in the prospective testing data set (eTable 4 in Supplement 1). The model performed with a comparable predictive accuracy with the senior surgeon (T: 72.51% [95% CI, 69.04%-75.78%] vs 71.08%; TL/L: 72.93% [95% CI, 69.48%-76.19%] vs 69.09%; mixed: 74.07% [95% CI, 70.66%-77.28%] vs 66.95%) and an increased accuracy compared with the junior surgeon (T: 65.24%; TL/L: 65.10%; mixed: 30.34%). An increased sensitivity was also found in detecting the curve types by the model compared with the senior surgeon (T: 82.31% [95% CI, 78.51%-85.70%] vs 76.64%; TL/L: 81.18% [95% CI, 77.29%-84.66%] vs 75.49%; mixed: 87.32% [95% CI, 82.10%-91.48%] vs 41.31%), as well as an increased NPV (T: 61.97% [95% CI, 56.45%-67.20%] vs 58.04%; TL/L: 62.11% [95% CI, 56.85%-67.11%] vs 55.56%; mixed: 92.52% [95% CI, 89.64%-94.65%] vs 75.35%). The junior surgeon’s sensitivity was 100% with 0% specificity and incomputable NPV due to all types being selected during manual assessment.

For distinguishing curve progressions in patients who had follow-ups using the app, the model had a predictive accuracy of 70.49% (95% CI, 57.43%-81.48%) and an AUC of 0.757 (95% CI, 0.630-0.858). The sensitivity was 63.33% (95% CI, 43.86%-80.87%), and the NPV was 68.57% (95% CI, 56.78%-78.37%). The experienced spine surgeon assessing progression and blinded to the radiographic examinations could distinguish the progression curves with an accuracy of 70.00%, sensitivity of 77.42%, and NPV of 72.00%. The junior surgeon stated they were incapable of distinguishing patients who had progressive deformity (Table 3).

**Table 3.** Performance Evaluation of ScolioNets on the Prospective Data Set in Predicting Disease Progression Using Back Photographs

Method	%					AUC
	Sensitivity	NPV	Specificity	PPV	Accuracy	
**ScolioNets**						
Progression	63.33 (43.86-80.87)	68.57 (56.78-78.37)	77.42 (58.90-90.41)	73.08 (57.25-84.62)	70.49 (57.43-81.48)	0.757 (0.630-0.858)
Nonprogression	77.42 (58.90-90.41)	73.08 (57.25-84.62)	63.33 (43.86-80.87)	68.57 (56.78-78.37)	70.49 (57.43-81.48)	0.757 (0.630-0.858)
**Senior surgeon**						
Progression	77.42	72.00	62.07	68.57	70.00	NA
Nonprogression	62.07	68.57	77.42	72.00	70.00	NA
**Junior surgeon** ^a^						
Progression	NA	NA	NA	NA	NA	NA
Nonprogression	NA	NA	NA	NA	NA	NA

Abbreviations: AUC, area under the receiver operating characteristic curve; NA, not available; NPV, negative predictive value; PPV, positive predictive value.

^a^ The junior surgeon stated was not able to distinguish whether the curve was progressing based on photographs of individuals’ backs.

### Model Interpretability

Score-CAM is a technique that helps visualize the focusing region of the proposed model. The interpretable heatmap reflects the areas in the image that are used to support classification decisions. With increased AIS severity, the attention pattern tended to have increased distortions (eFigure 8 in Supplement 1). For curve typing, we found that T curves had attention in the T region whereas L curves had attention in the L region (eFigure 8 in Supplement 1). Mixed curves had attention in both T and L regions (eFigure 8 in Supplement 1).

## Discussion

In this diagnostic study, considering the growing role of digital health and out-of-hospital management and monitoring for AIS, we developed the AlignProCARE open platform app,^18^ powered by the ScolioNets deep learning model, to automatically classify AIS severities and curve types without the need for specific backgrounds or medical equipment. Compared with existing products^34,35^ for AIS monitoring, our platform has more diverse and comprehensive features, with wide system support that is more user-centric. Considering the decisions made by spine specialists using radiographs as GT, the prospective results achieved by the model had comparable or superior sensitivity and NPV with spine surgeons visually assessing the unclothed back photos of individuals with AIS. When attempting to differentiate patients requiring no interventions, both senior and junior surgeons encountered difficulties in making a decision solely based on unclothed backs, and the model demonstrated superior performance compared with both surgeons. For the performance of curve type identification, the junior surgeon was not able to identify the curve type only based on the unclothed back photos; therefore, all the curve types were recommended to be checked with physical examination.

In distinguishing disease progression based on 2 unclothed back photos, the model outperformed specialists. In assessing the model’s performance, sensitivity and NPV are particularly clinically relevant because it is important to promptly detect any disease progression to trigger early interventions but also avoid unnecessary interventions. In training the model, we considered the spine specialists’ classifications of the scoliosis as the main task and set a smaller weight for the type of scoliosis; thus, the severity classification outcomes were superior. In practice, the proposed platform could provide automated identification of disease severity for patients with scoliosis. Furthermore, for individuals with no or mild scoliosis (Cobb angle <20°), the model may potentially decrease the requirement for radiographic screening, leading to improved health outcomes for patients. However, for patients detected as having moderate or severe AIS, radiographic examinations are still required for more precise evaluation.

Surgical procedures to treat AIS are among the most costly interventions for children in hospitals,^36,37^ and progressive deterioration during puberty occurs in two-thirds of patients.^38^ Thus, close monitoring is important. Previous photography-based attempts^39^ to assess AIS based on back appearance features were found to be unreliable for various features that characterize scoliosis, since the traditional algorithms struggle to extract distinguishable features from back images. Deep learning is recognized as a promising approach due to its powerful ability to extract distinguishable features automatically. As radiographic and back photographic appearance had visual feature associations in both severity and curve type assessment, a 2019 study^17^ explored deep learning to facilitate image-based examinations for back malformations using retrospectively collected standardized images captured in a controlled environment, with no prospective validations. In our study, we first improved the severity classification with clinically meaningful information recommending follow-up or considering surgery, and we achieved improved performance by introducing attention blocks in the model. We further explored the single back image–based deep learning classification of curve types and disease progressions, which are challenging tasks for spine specialists without radiographs. Lastly, we deployed the prospectively validated model to an open platform for other clinicians and researchers to use to assist in monitoring of patients with scoliosis, identifying disease progression, and facilitating efficient patient call-back. It has the potential to provide remote scoliosis assessment for individuals at risk of curve progression and individuals living in places where an experienced spine surgeon is not readily accessible, eg, low-resource countries and regions.

### Limitations

This study has some limitations. The training data set was collected using the 3 most common types of smartphone devices in the local region; further assessment is needed to determine whether performance will change with the use of other image acquisition devices. It is worth noting that further image preprocessing was conducted to standardize the input images for the model within the platform. Considering the features of participants with obesity may not be distinguishable enough for our model, no patients with BMI greater than 30 were recruited in this study. Furthermore, to test the robustness of the model, we used the collected raw prospective data. Unlike a previous study,^17^ we did not resample the prospective cohort to match the data distribution of the training data set. Thus, this prospective validation trial demonstrates the adequacy of the model for the classification task of scoliosis. However, the prospective study was still performed in our regional health care system. A multicenter trial with international collaborators to further evaluate the robustness of the model is needed.

## Conclusions

The findings of this diagnostic study suggest that the AlignProCARE app powered by the ScolioNets deep learning model could provide accessible mobile assessments of AIS, especially for patients with barriers to access care from experienced spine specialists. With no extra radiation and minimal cost, this model could provide continuous monitoring with prompt interventions triggered when progression is detected. Our open platform has the benefits of lower risk and low-cost, easy access. This could contribute to further treatment planning and monitoring for the patient by providing computer-aided real-time assessments to aid physicians’ management decision-making. In the future, the open platform could continuously benefit spine specialists and patients internationally by providing a fully automated, fast, unbiased, and comprehensive analysis of spine malalignments.

## Notes

Supplement 1. **eAppendix 1.** Inclusion and Exclusion Criteria **eAppendix 2.** Definitions of AIS Severity and Curve Type **eAppendix 3.** Open Platform for AIS Evaluations **eAppendix 4.** Multilayer CNNs and Model Selection **eAppendix 5.** The Attention Algorithms and Multitasking Strategy **eAppendix 6.** Development of ScolioNets for Comprehensive AIS Analysis **eFigure 1.** Overview of the Data and the Proposed Deep Learning Diagnosis Flow **eFigure 2.** Use of ScolioNets Within the AlignProCARE Platform **eFigure 3.** Paired Samples of Radiographs and Bare Back Images **eFigure 4.** ROC Curves and Confusion Matrices for Severity Classification in the In-House Validation Tests of Different Models **eFigure 5.** The Detailed Architecture of ScolioNets for Severity Grading, Curve Type Classification, and Follow-Up Progression Prediction **eFigure 6.** Radiographs With Confirmed No or Mild, Moderate, and Severe Classifications in Cohorts 1 and 2 **eFigure 7.** Radiographs Taken for Each Severity Class and Curve Type **eFigure 8.** Heatmaps of Areas Used to Support Classification Decisions **eTable 1.** Comparative Results for In-House Validation Set **eTable 2.** Significance Level Results **eTable 3.** Comparison of the Performance Evaluation Metrics Between ScolioNets and Surgeons on the Prospective Dataset in Distinguishing Severe Curves for Surgical Intervention Consideration Using Single Back Photographs **eTable 4.** Comparison of the Performance Evaluation Metrics Between ScolioNets and Surgeons on the Prospective Dataset in Distinguishing Curve Types Using Single Back Photographs
eReferences.

Supplement 2.
Data Sharing Statement

## References

[1] de Sèze M, Cugy E. Pathogenesis of idiopathic scoliosis: a review. Ann Phys Rehabil Med. 2012;55(2):128-138. doi:10.1016/j.rehab.2012.01.003 22321868

[2] Fong DY, Cheung KM, Wong YW, . A population-based cohort study of 394,401 children followed for 10 years exhibits sustained effectiveness of scoliosis screening. Spine J. 2015;15(5):825-833. doi:10.1016/j.spinee.2015.01.019 25615844

[3] Luk KD, Lee CF, Cheung KM, . Clinical effectiveness of school screening for adolescent idiopathic scoliosis: a large population-based retrospective cohort study. Spine (Phila Pa 1976). 2010;35(17):1607-1614. doi:10.1097/BRS.0b013e3181c7cb8c 20453727

[4] Wong JSH, Cheung KMC. Impact of COVID-19 on orthopaedic and trauma service: an epidemiological study. J Bone Joint Surg Am. 2020;102(14):e80. doi:10.2106/JBJS.20.00775 32675668 PMC7431143

[5] Brinjikji W, Luetmer PH, Comstock B, . Systematic literature review of imaging features of spinal degeneration in asymptomatic populations. AJNR Am J Neuroradiol. 2015;36(4):811-816. doi:10.3174/ajnr.A4173 25430861 PMC4464797

[6] Del Grande F, Maus TP, Carrino JA. Imaging the intervertebral disk: age-related changes, herniations, and radicular pain. Radiol Clin North Am. 2012;50(4):629-649. doi:10.1016/j.rcl.2012.04.014 22643389

[7] Melhem E, Assi A, El Rachkidi R, Ghanem I. EOS biplanar x-ray imaging: concept, developments, benefits, and limitations. J Child Orthop. 2016;10(1):1-14. doi:10.1007/s11832-016-0713-0 26883033 PMC4763151

[8] Zhang T, Li Y, Cheung JPY, Dokos S, Wong KY-K. Learning-based coronal spine alignment prediction using smartphone-acquired scoliosis radiograph images. IEEE Access. 2021;9:38287-38295. doi:10.1109/ACCESS.2021.3061090

[9] Chen B, Xu QH, Wang LS, Leung S, Chung J, Li S. An automated and accurate spine curve analysis system. IEEE Access. 2019;7:124596-124605. doi:10.1109/ACCESS.2019.2938402

[10] Horng MH, Kuok CP, Fu MJ, Lin CJ, Sun YN. Cobb angle measurement of spine from x-ray images using convolutional neural network. Comput Math Methods Med. 2019;2019:6357171. doi:10.1155/2019/6357171 30996731 PMC6399566

[11] Levy AR, Goldberg MS, Hanley JA, Mayo NE, Poitras B. Projecting the lifetime risk of cancer from exposure to diagnostic ionizing radiation for adolescent idiopathic scoliosis. Health Phys. 1994;66(6):621-633. doi:10.1097/00004032-199406000-00002 8181937

[12] Doody MM, Lonstein JE, Stovall M, Hacker DG, Luckyanov N, Land CE. Breast cancer mortality after diagnostic radiography: findings from the U.S. Scoliosis Cohort Study. Spine (Phila Pa 1976). 2000;25(16):2052-2063. doi:10.1097/00007632-200008150-00009 10954636

[13] Bone CM, Hsieh GH. The risk of carcinogenesis from radiographs to pediatric orthopaedic patients. J Pediatr Orthop. 2000;20(2):251-254. doi:10.1097/01241398-200003000-00023 10739292

[14] Goldberg MS, Mayo NE, Levy AR, Scott SC, Poîtras B. Adverse reproductive outcomes among women exposed to low levels of ionizing radiation from diagnostic radiography for adolescent idiopathic scoliosis. Epidemiology. 1998;9(3):271-278. doi:10.1097/00001648-199805000-00010 9583418

[15] Weinstein SL, Dolan LA, Spratt KF, Peterson KK, Spoonamore MJ, Ponseti IV. Health and function of patients with untreated idiopathic scoliosis: a 50-year natural history study. JAMA. 2003;289(5):559-567. doi:10.1001/jama.289.5.559 12578488

[16] Choi R, Watanabe K, Jinguji H, . CNN-based spine and cobb angle estimator using moire images. IIEEJ Trans Image Electronics Visual Computing. 2017;5(2):135-144. doi:10.11371/tievciieej.5.2_13535337274

[17] Yang J, Zhang K, Fan H, . Development and validation of deep learning algorithms for scoliosis screening using back images. Commun Biol. 2019;2:390. doi:10.1038/s42003-019-0635-8 31667364 PMC6814825

[18] Meng N, Cheung JPY, Wong KK, . An artificial intelligence powered platform for auto-analyses of spine alignment irrespective of image quality with prospective validation. EClinicalMedicine. 2022;43:101252. doi:10.1016/j.eclinm.2021.101252 35028544 PMC8741432

[19] Yoon JW, Welch RL, Alamin T, . Remote virtual spinal evaluation in the era of COVID-19. Int J Spine Surg. 2020;14(3):433-440. doi:10.14444/7057 32699768 PMC7343271

[20] Chung N, Cheng YH, Po HL, . Spinal phantom comparability study of Cobb angle measurement of scoliosis using digital radiographic imaging. J Orthop Translat. 2018;15:81-90. doi:10.1016/j.jot.2018.09.005 30533384 PMC6258248

[21] Nachemson AL, Peterson LE. Effectiveness of treatment with a brace in girls who have adolescent idiopathic scoliosis: a prospective, controlled study based on data from the Brace Study of the Scoliosis Research Society. J Bone Joint Surg Am. 1995;77(6):815-822. doi:10.2106/00004623-199506000-00001 7782353

[22] Eyvazov K, Samartzis D, Cheung JP. The association of lumbar curve magnitude and spinal range of motion in adolescent idiopathic scoliosis: a cross-sectional study. BMC Musculoskelet Disord. 2017;18(1):51. doi:10.1186/s12891-017-1423-6 28143455 PMC5282845

[23] Wang H, Zhang T, Cheung KM, Shea GK. Application of deep learning upon spinal radiographs to predict progression in adolescent idiopathic scoliosis at first clinic visit. EClinicalMedicine. 2021;42:101220. doi:10.1016/j.eclinm.2021.101220 34901796 PMC8639418

[24] Katz DE, Herring JA, Browne RH, Kelly DM, Birch JG. Brace wear control of curve progression in adolescent idiopathic scoliosis. J Bone Joint Surg Am. 2010;92(6):1343-1352. doi:10.2106/JBJS.I.01142 20516309

[25] Lin S, Yang L, Saleemi I, Sengupta S. Robust high-resolution video matting with temporal guidance. Paper presented at: Institute of Electrical and Electronics Engineers and Computer Vision Foundation Winter Conference on Applications of Computer Vision; January 3-7, 2021; Waikoloa, HI.

[26] Sokolova M, Lapalme G. A systematic analysis of performance measures for classification tasks. Inf Process Manage. 2009;45(4):427-437. doi:10.1016/j.ipm.2009.03.002

[27] Wong TT. Linear approximation of F-measure for the performance evaluation of classification algorithms on imbalanced data sets. IEEE T Knowl Data En. 2022;34(2):753-763. doi:10.1109/TKDE.2020.2986749

[28] Niu ZY, Zhong GQ, Yu H. A review on the attention mechanism of deep learning. Neurocomputing. 2021;452:48-62. doi:10.1016/j.neucom.2021.03.091

[29] Fukui H, Hirakawa T, Yamashita T, Fujiyoshi H. Attention branch network: learning of attention mechanism for visual explanation. *arXiv*. Preprint posted online December 25, 2018. doi:10.1109/CVPR.2019.01096

[30] Zhou B, Khosla A, Lapedriza A, Oliva A, Torralba A. Learning deep features for discriminative localization. *arXiv*. Preprint posted online December 14, 2015. doi:10.1109/CVPR.2016.319

[31] Desai S, Ramaswamy HG. Ablation-CAM: visual explanations for deep convolutional network via gradient-free localization. Institute of Electrical and Electronics Engineers Winter Conference on Applications of Computer Vision; March 1-5, 2020; Snowmass, CO. doi:10.1109/WACV45572.2020.9093360.

[32] Jalwana MAAK, Akhtar N, Bennamoun M, Mian A. CAMERAS: enhanced resolution and sanity preserving class activation mapping for image saliency. arXiv. Preprint posted online June 20, 2021. doi:10.1109/CVPR46437.2021.01606

[33] Wang H, Wang Z, Du M, . Score-CAM: score-weighted visual explanations for convolutional neural networks. *arXiv*. Preprint posted online October 3, 2019. doi:10.1109/CVPRW50498.2020.00020/arXiv.1910.01279

[34] van West HM, Herfkens J, Rutges JPHJ, Reijman M. The smartphone as a tool to screen for scoliosis, applicable by everyone. Eur Spine J. 2022;31(4):990-995. doi:10.1007/s00586-021-06860-x 34008090

[35] Franko OI, Bray C, Newton PO. Validation of a scoliometer smartphone app to assess scoliosis. J Pediatr Orthop. 2012;32(8):e72-e75. doi:10.1097/BPO.0b013e31826bb109 23147635

[36] Raval MV, Reiter AJ, McCarthy IM. Association of children’s hospital status with value for common surgical conditions. JAMA Netw Open. 2022;5(6):e2218348. doi:10.1001/jamanetworkopen.2022.18348 35749117 PMC9233238

[37] Gill PJ, Thavam T, Anwar MR, ; Ontario Pediatric Hospital Care Study Group (OPHCSG) and the Canadian Paediatric Inpatient Research Network (PIRN). Prevalence, cost, and variation in cost of pediatric hospitalizations in Ontario, Canada. JAMA Netw Open. 2022;5(2):e2147447. doi:10.1001/jamanetworkopen.2021.47447 35138399 PMC8829658

[38] Cheng JC, Castelein RM, Chu WC, . Adolescent idiopathic scoliosis. Nat Rev Dis Primers. 2015;1:15030. doi:10.1038/nrdp.2015.30 27188385

[39] Akazawa T, Torii Y, Ueno J, Saito A, Niki H. Mobile application for scoliosis screening using a standard 2D digital camera. Cureus. 2021;13(3):e13944. doi:10.7759/cureus.13944 33880282 PMC8051536

