# Supplementary material for: Deep Learning Model to Classify and Monitor Idiopathic Scoliosis in Adolescents Using a Single Smartphone Photograph
Source: JAMA Netw Open. 2023 Aug 23;6(8):e2330617. doi: 10.1001/jamanetworkopen.2023.30617 (PMC10448299; doi:10.1001/jamanetworkopen.2023.30617)
Supplement: Supplement 1. — eAppendix 1. Inclusion and Exclusion Criteria eAppendix 2. Definitions of AIS Severity and Curve Type eAppendix 3. Open Platform for AIS Evaluations eAppendix 4. Multilayer CNNs and Model Selection eAppendix 5. The Attention Algorithms and Multitasking Strategy eAppendix 6. Development of ScolioNets for Comprehensive AIS Analysis eFigure 1. Overview of the Data and the Proposed Deep Learning Diagnosis Flow eFigure 2. Use of ScolioNets Within the AlignProCARE Platform eFigure 3. Paired Samples of Radiographs and Bare Back Images eFigure 4. ROC Curves and Confusion Matrices for Severity Classification in the In-House Validation Tests of Different Models eFigure 5. The Detailed Architecture of ScolioNets for Severity Grading, Curve Type Classification, and Follow-Up Progression Prediction eFigure 6. Radiographs With Confirmed No or Mild, Moderate, and Severe Classifications in Cohorts 1 and 2 eFigure 7. Radiographs Taken for Each Severity Class and Curve Type eFigure 8. Heatmaps of Areas Used to Support Classification Decisions eTable 1. Comparative Results for In-House Validation Set eTable 2. Significance Level Results eTable 3. Comparison of the Performance Evaluation Metrics Between ScolioNets and Surgeons on the Prospective Dataset in Distinguishing Severe Curves for Surgical Intervention Consideration Using Single Back Photographs eTable 4. Comparison of the Performance Evaluation Metrics Between ScolioNets and Surgeons on the Prospective Dataset in Distinguishing Curve Types Using Single Back Photographs eReferences. [file jamanetwopen-e2330617-s001.pdf]

Supplemental Online Content

Zhang T, Zhu C, Zhao Y, et al. Deep learning model to classify and monitor idiopathic scoliosis in adolescents using a single smartphone photograph. *JAMA Netw Open*. 2023;6(8):e2330617. doi:10.1001/jamanetworkopen.2023.30617

eAppendix 1. Inclusion and Exclusion Criteria
eAppendix 2. Definitions of AIS Severity and Curve Type
eAppendix 3. Open Platform for AIS Evaluations
eAppendix 4. Multilayer CNNs and Model Selection
eAppendix 5. The Attention Algorithms and Multitasking Strategy
eAppendix 6. Development of ScolioNets for Comprehensive AIS Analysis
eFigure 1. Overview of the Data and the Proposed Deep Learning Diagnosis Flow
eFigure 2. Use of ScolioNets Within the AlignProCARE Platform
eFigure 3. Paired Samples of Radiographs and Bare Back Images
eFigure 4. ROC Curves and Confusion Matrices for Severity Classification in the In-House Validation Tests of Different Models
eFigure 5. The Detailed Architecture of ScolioNets for Severity Grading, Curve Type Classification, and
eFigure 6. Radiographs With Confirmed No or Mild, Moderate, and Severe Classifications in Cohorts 1 and 2
eFigure 7. Radiographs Taken for Each Severity Class and Curve Type
Follow-Up Progression Prediction
eFigure 8. Heatmaps of Areas Used to Support Classification Decisions
eTable 1. Comparative Results for In-House Validation Set
eTable 2. Significance Level Results
eTable 3. Comparison of the Performance Evaluation Metrics Between ScolioNets and Surgeons on the Prospective Dataset in Distinguishing Severe Curves for Surgical Intervention Consideration Using Single Back Photographs
eTable 4. Comparison of the Performance Evaluation Metrics Between ScolioNets and Surgeons on the Prospective Dataset in Distinguishing Curve Types Using Single Back Photographs
eReferences.

This supplemental material has been provided by the authors to give readers additional information about their work.

© 2023 Zhang T et al. *JAMA Network Open.*

eAppendix 1. Inclusion and Exclusion Criteria

Inclusion criteria

1) Males and females who were older than 10 years with AIS, low back pain, or degenerative malformation were included.
2) Participants or the guardian of the participants (if the participants were under 18) could complete the written informed consent (in either English or Chinese versions).
3) Able to understand the nature and scope of the study.

Exclusion criteria

1) Patients were excluded if they were diagnosed with or have any signs of psychological disorders that might influence the compliance of the study.
2) Patients were excluded if they had any pre-diagnosed systematic neural disorders that might influence the mobility of the patients (e.g. prior cerebrovascular accident, Parkinson's disease, myopathy).
3) Patients were excluded if they had the following musculoskeletal diseases:
    - Congenital spinal malformation,
    - McCune-Albright syndrome,
    - Early-onset scoliosis,
    - Previous spine operations and instrumentation performed,
    - Trauma that might impair posture and mobility.
4) Patients were excluded if they had any oncological diseases.
5) Patients were excluded if they had severe skin disorders and/or lesions at the back.
6) Patients were excluded if they had any other systematic diseases.
7) Patients were excluded if the patient could not complete the consent process.
8) Patients were excluded if the patient and the carer did not have access to smartphones, and/or unable to attend training sessions for using the mobile platform.
9) Patients were excluded if the BMI was over 30.

eAppendix 2. Definitions of AIS Severity and Curve Type

To measure the Cobb angle (CA), the end vertebrae (the most tilted vertebrae from the horizontal apical vertebra) was identified at the upper and lower ends of the curve, and the angle formed by lines drawn at the superior and inferior endplates of the upper and lower end vertebrae respectively is measured as the CA (eFigure 3). Different clinical interventions are applied for different AIS severities. We grouped normal and mild cases into one category as this group of individuals do not require specialist care and screening programmes suggest specialist referral only with CA>20°. Moderate cases recommend for non-surgical interventions with regular FUs, and severe cases should be considered for surgical intervention.

The majority of population have 12 thoracic vertebrae and 5 lumbar vertebrae. if the apex locates between the 1st and the 11th thoracic vertebrae it is considered as the thoracic curve (T: including T11-12 disc), whereas if the apex locates between the 12th thoracic and the 5th lumbar vertebrae it is considered as the thoracolumbar/lumbar curve (TL/L). This curve type classification is commonly used in clinical practice.[1] Complex curve type classifications including Lenke or King classifications were not used, because these classifications are relevant to surgical planning rather than out-of-hospital assessments.

eAppendix 3. Open Platform for AIS Evaluations

© 2023 Zhang T et al. *JAMA Network Open.*

We developed our model on ordinary back images with the gold standard disease severity taxonomy (i.e., GTs generated from the X-ray images reviewed by spine surgeons) to get accurate and effective features of the spine specialist-level classification. Specifically, we randomly divided cohort 1 by 8:2, corresponding to the training set and the in-house validation set. The in-house validation set was used to evaluate and select different deep learning models. Data augmentation methods[2-4] including random rotate (-10°~10°), affine transform, crop and pad, gaussian blur, sharpen, change contrast and brightness were introduced to cohort 1. Each method was set to appear with a 50% probability and combined them for each image of cohort 1.

Overview of the AlignProCARE platform (available via web application, App Store and Google Play) powered by *ScolioNets* for classifying malformation severities and curve types using radiation-free back images (eFigure 2) is to exemplify the usefulness of *ScolioNets*. It integrates the smartphone photos, radiographs and GT disease taxonomy to predict clinical interventions (both for severity levels and disease types) of AIS patients, which is made freely open for all clinicians and researchers doing research on AIS. For user convenience, an example (eFigure 2) is provided. Entering the platform, users can manage the patient information along with back images and/or radiographs. Cases can be added by clicking the 'Add patient' button and images can be taken by clicking the 'Upload photo' button. The back images are sent to the server located in our hospital for automated processing. Results are sent back to the mobile device for interpretable analysis with auto-results, with the flexibility for the linked clinician to modify and confirm. If the modified landmarks are not saved, the CNN computed results are saved in the system until further updates. All images are securely and anonymously stored with authorized access under local regulation for research purposes.

eAppendix 4. Multilayer CNNs and Model Selection

Several deep convolutional network benchmarks were compared using the data from cohort 1 (eTable 1). In our CNN framework, there were 1 input layer, 84 hidden layers, and 1 output layer. In the input layer, the input image was processed by convolution, batch normalization (BN) and pooling operations, to reduce the interference caused by the difference in the range of values of the input data in each dimension.

In the hidden layers, neurons were in each layer and were connected and propagated containing internal feature coding and computational outcome. The convolutional layers were used for feature extraction and presentation, and a commonly used rectified linear unit (ReLU) function was selected to activate the outcome of a neuron and defined as follows:

$$y = \max(0, x)$$

where *x* was the weighted sum of a neuron and *y* was the output of the activation function.

In the output layer, the weights of all neurons' output from the hidden layer were fully connected to obtain the required probability values for classification. Since the final output of the classification task was essentially the probability value of the image input in each category, a softmax function was generally introduced at the end in order to map the output of the fully connected layer to the (0,1) range with a summation value of 1, which is calculated as follows:

$$S_i = \frac{e^{V_i}}{\sum_j e^{V_j}}$$

where V was the input of the softmax layer derived from the fully connected layer and S was the final output. In the models for back image-based severity and type prediction, S_i was a value in the range 0-1 representing the probability of a back photo classified as normal-mild, moderate, or severe with T, TL/L, or mixed type.

For the experiment on model selection, we trained five models, Resnet50[5], Densenet169[6], VGG16[7],

InceptionV4[8] and ResNeXt50[9], of which results are shown in eFigure 4 and eTable 2, where the model with Resnet50 and Densenet169 as backbones performed relatively better. Our initial selection of models was based on the AUC metric to judge the comprehensive performance of the models, and Resnet50 and Densenet169 performed best on the macro-average AUC metric for the severity classification task (eTable 2). Although, InceptionV4 was comparable to Resnet50 and Desnsenet169 in many metrics in both FU and CS statistics (eTable 2), we finally chose Resnet50 and Densenet169 as the backbone considering the size of the model. To further improve performance, we added multi-tasking strategy and attention.

eAppendix 5. The Attention Algorithms and Multitasking Strategy

The human visual system tends to focus on a certain part of the image that is useful for judgment and ignore other unimportant areas. The attention mechanism[10,11] is a method that allows a network to mimic the human visual system and tend to pay attention to a certain part of the image when performing a classification task. Multi-task strategy[12-14] is designed to improve the model performance by learning multiple tasks in parallel so that the results can interact with each other, either by sharing the weights of feature extraction on the network or by interacting only on some key layers, and finally using classifiers to classify each task.

Different attention algorithms were tested, including SE bloc [15] and residual attention block[16]. Empirically, residual attention block with the activation function for channel and spatial mixed attention performed the best. The activation function is as follows:

$$f(x_{i,c}) = \frac{1}{1 + e^{-x_{i,c}}}$$

where *x* was the weighted sum of a neuron, *i* was position of the feature map, *c* was channel of the feature map.

Previous studies reported[16] the residual attention block is an improvement on the residual net, thus we added the attention module to both Resnet50 and Densenet169 for further testing. Resnet50 and Densnet169 consist of 4 residual blocks and 4 dense blocks respectively, so we added a total of 4 attention blocks after the input layer and after each submodule respectively. With attention introduced, we empirically discovered that spatial and channel mixed attention improved the performance of the classification task. The model with Resnet50 as backbone with attention module performed better and then was selected to be our final classification model *ScolioNets*.

Multi-task strategy was tested in addition to the attention to try to improve the generalization ability of the model. We did not change the design of the feature extraction part of the network, shared the feature extraction weights for the severity and type of scoliosis tasks, and only used 3 parallel classifiers for each task at the end. Since our main task is the severity and type classification task, we set the loss to the sum of the three-classification problem loss and set the weight of the type of scoliosis problem to 0.5.

After the selection, our *ScolioNets* consisted of 1 input layer, 84 hidden layers and 1 output layer, where the hidden layers consisted of 4 residual blocks and 3 attention modules. In the output layer, we used three parallel FC layers to implement multi-task method to finish the classification of the severity, while for classification of type of curve, we only used one FC layer.

eAppendix 6. Development of ScolioNets for Comprehensive AIS Analysis

ScolioNets comprises four steps, including the radiation-free back photo-based severity auto-classification, the radiation-free back photo-based disease typing, the FUs progression prediction, and intervention recommendations (eFigure 1). We detected the normal-mild malformations and the severe malformations based on the GT X-rays analytical results, to avoid unnecessary radiation to the patients and

to activate prompt interventions. The curves were typed into the single curve with T or TL/L, or mixed curves with more than one curve, manually by spine specialists on radiographs via consensus. To enable back image-based predictions, we implemented a deep learning framework *ScolioNets* with ResNet50[5] as the backbone (eFigure 5). The original ResNet50 contains 50 layers and 25.5×10^6 parameters. To increase the robustness of the model and reduce its complexity, we modified the model by adding residual attention blocks[16] (eFigure 5) and auxiliary classification branches, with an additional attention module and multi-task strategies. *ScolioNets* framework outperformed other deep learning models (Densenet169, ResNet50, ResNeXt50, Vgg16, and Inception V4) when we tested for Spine specialists-level classification of scoliosis (eTable 2). The FUs progression was predicted by two images taken from one person at different times.

During model development our in-house validation set consisted of 351 images. We performed data augmentation on the validation sets to be proportionally matching with the training dataset. The parameter optimization was stopped when the performance AUC was not improving. The validation set was used for model selection during training. The in-house dataset was not used for training, but for the in-house performance validation of the models, before a prospective testing study. The prediction of each class performed well with an AUC of around 0.85. All proposed models performed similarly and the ROC curve of *ScolioNets* had the most balanced performance on each classification task (eFigure 4, eTable 2).

For model training, we used a workstation configured with an Intel(R) Xeon(R) Gold 5218 2.30GHz central processing unit, 308 GB of RAM, a NVIDIA GeForce GTX 300 core and PyTorch. Models were trained by minimizing the general cross-entropy loss function between predictions and GT labels,

$$Loss = -(y \log \hat{y} + (1-y)\log(1-\hat{y})]$$

where y was the GT and $\hat{y}$ was the prediction.

During training SGD optimizer in PyTorch was adopted and a decay factor γ was used to control the learning rate at each epoch as described below:

$$lr_{new} = lr_{initial} * \gamma^{epoch//stepsize}$$

where $lr_{initial}$ was the initial learning rate, lr_{new} was the learning rate at i^{th} epoch and step size was the learning rate decay step. Adjustable parameters such as initial rate, decay and batch size were simultaneously adjusted to improve the model performance. Our initial learning rate of 0.01, the momentum of 0.9, weight decay of 0.001, γ of 0.1 and step size of 20, batch size 16 and epoch of 50.

The *ScolioNets* achieved an AUC score of 0.881 on the in-house validation set, details shown in eTable 2. First, 1,780 manually labelled radiographic results of the disease severity and curve type were used as GT for the paired smartphone photos. From the ROC curve (eFigure 4, eTable 2), *ScolioNets* achieved an AUC value of 0.869 in distinguishing normal-mild cases from other cases with back photos recommending no clinical intervention, and an AUC value of 0.958 in predicting severe cases recommending possible surgical interventions (eFigure 4, eTable 2). To train models for curve type predictions, 1,780 back photos with a single T curve, a single TL/L curve or mixed curves were used from cohort 1 after eliminating the normal cases with no curves.

© 2023 Zhang T et al. *JAMA Network Open.*

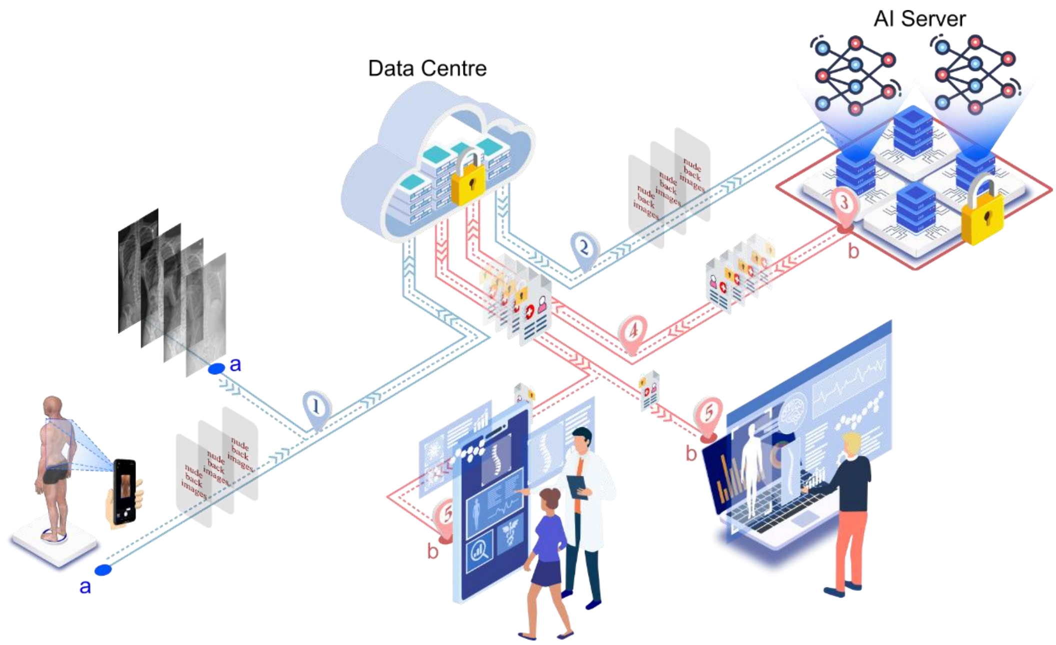

eFigure 1. Overview of the Data and the Proposed Deep Learning Diagnosis Flow

a, Collection of the dataset. The training dataset was composed of the captured phone images from the involved patients which were securely stored in our data centre. To obtain the malformation severity for each patient, radiographs were also scanned, and further analysed by the specialists. The diagnosis results, including the Cobb angles and the curve types, were used as the ground truth labels for the collected phone images. **b,** Online AIS checking flow. The proposed multi-task attention-based CNN model (*ScolioNets*) was deployed on the backend AI server after training for further prospective testing. Practically, AIS checking could be conducted in steps: 1) back photographs are captured and transferred to the data centre together with X-ray images; 2) the encrypted and deidentified back photographs are sent to the AI server for diagnosis via the network; 3) the diagnosing server performs the classification of AIS severity and curve types; 4) the diagnosing results are returned to the patients through the network; 5) the analytic results are visualized at the client end equipment, such as the smartphone and laptop computer.

© 2023 Zhang T et al. *JAMA Network Open.*

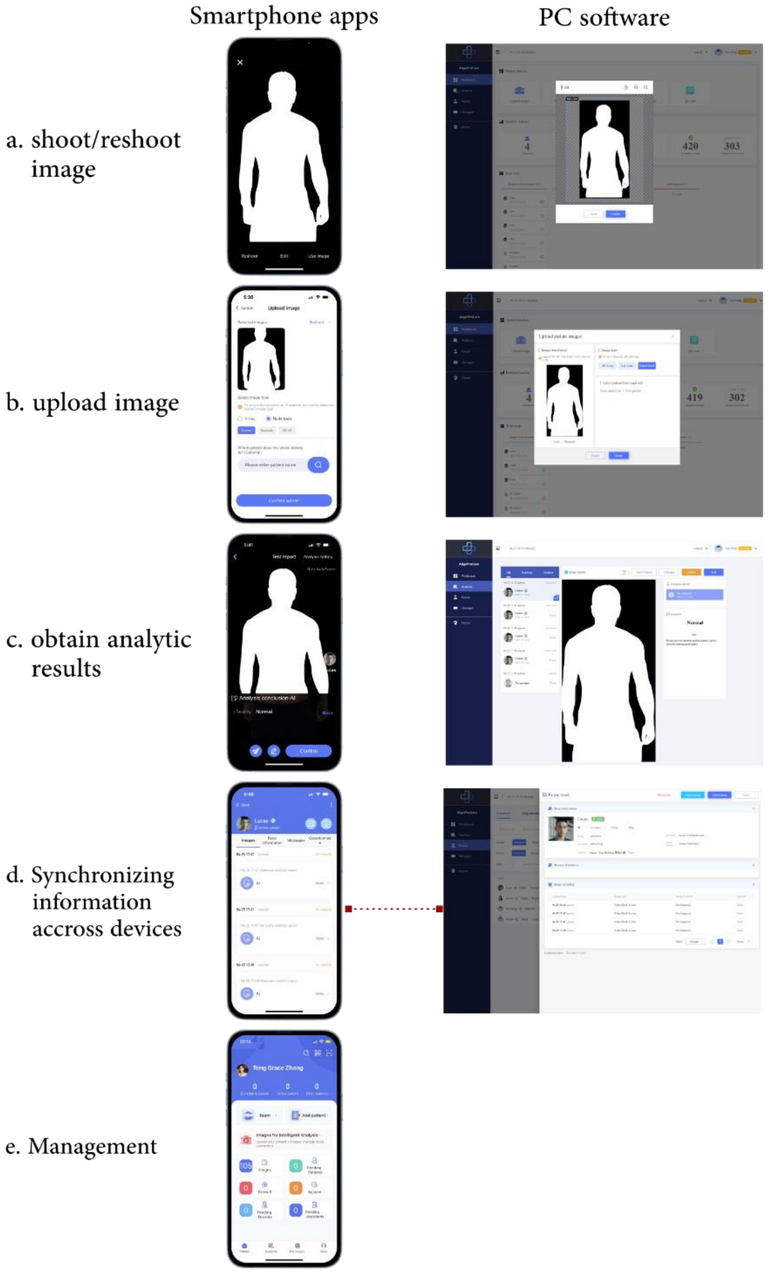

eFigure 2. Use of ScolioNets Within the AlignProCARE Platform

It integrates the smartphone photos, radiographs and GT disease taxonomy to predict clinical interventions (both for different severity levels and disease types) of AIS patients, which is made freely open for all clinicians and researchers doing research on AIS. Entering the platform, nude back images can be uploaded and auto-analyzed. Cases can be added by clicking 'Add patient' button, images can be taken by clicking the 'Upload Images' (a), and the image can be sent to the AI server in our lab for automated analysis by clicking 'Confirm submit' button (b). The results are sent back to devices for viewing and/or further amendments (c). Registered users can have their images synchronized between smartphone apps and PC software (d). Doctors can communicate and refer patients among their team, as well as manage patients attending different clinics easily (e). The human unclothed back images are replaced by the human silhouette images.

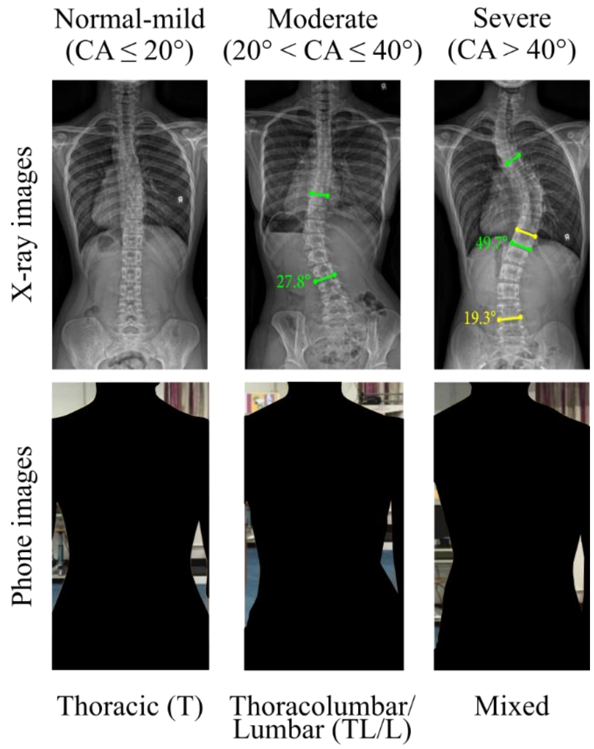

eFigure 3. Paired Samples of Radiographs and Bare Back Images

First row: Classifications for AIS severity (normal-mild, moderate and severe) and curve types (T, TL/L, and mixed) on radiographs. Second row: The examples for the corresponding bareback images. The human unclothed back images are replaced by the human silhouette images.

© 2023 Zhang T et al. *JAMA Network Open.*

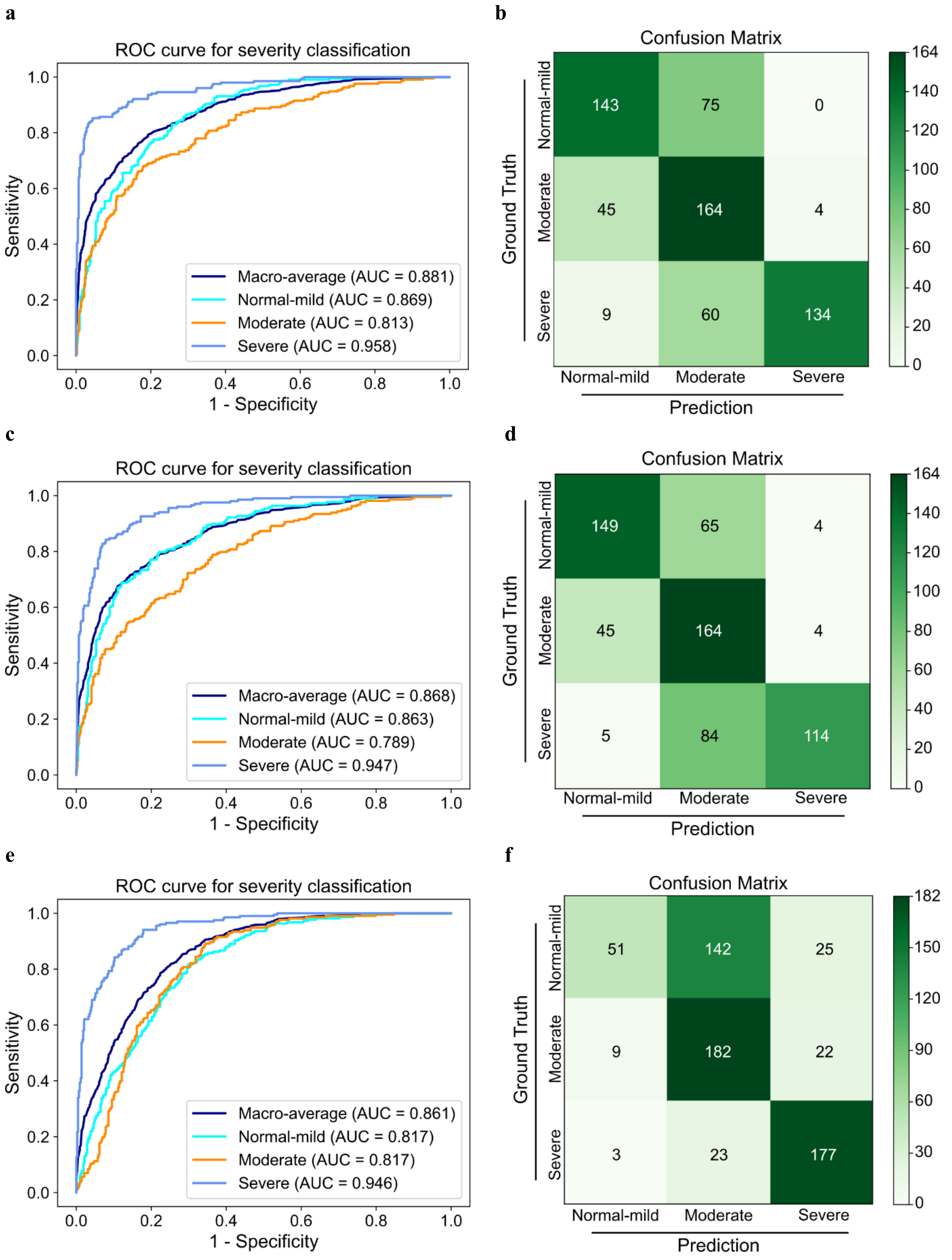

© 2023 Zhang T et al. *JAMA Network Open.*

g

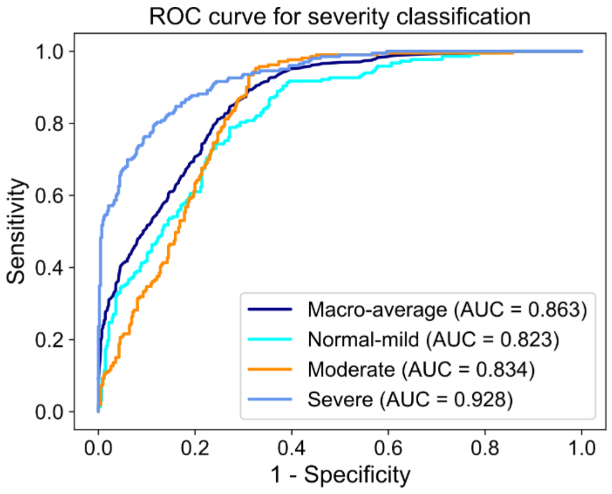

h

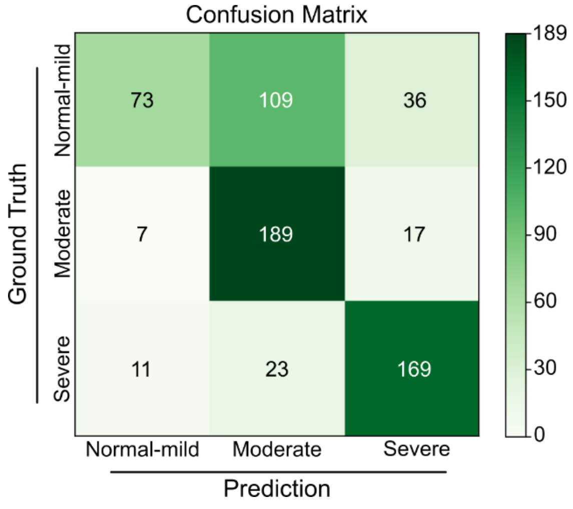

i

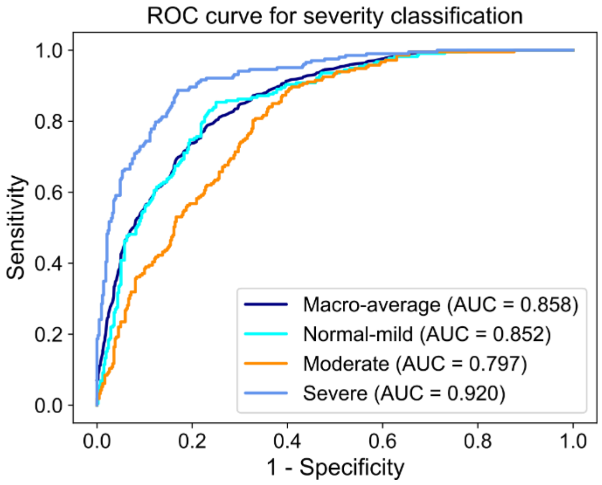

j

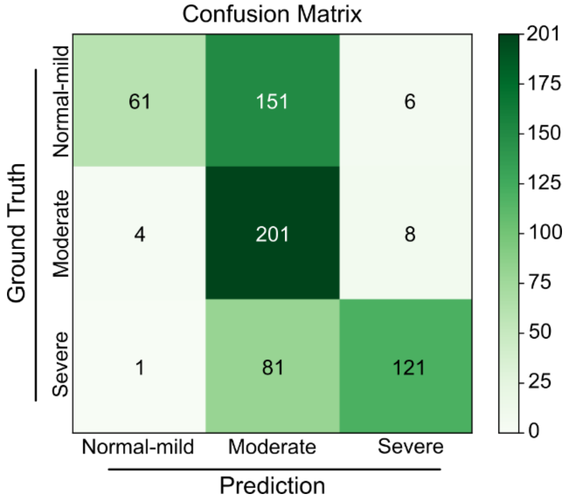

k

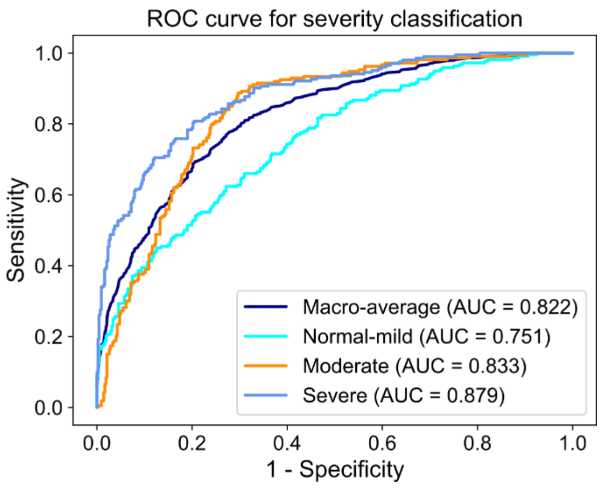

l

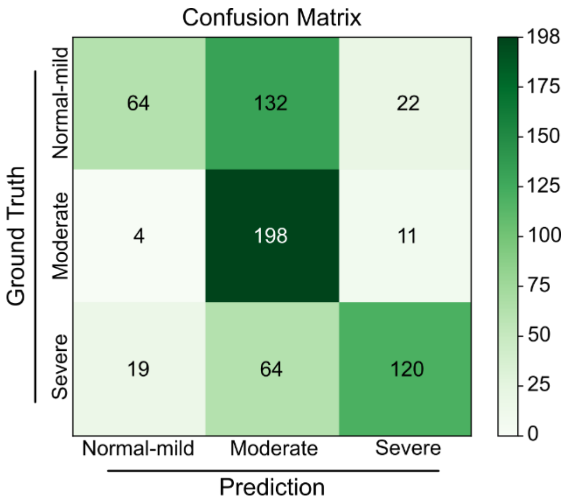

© 2023 Zhang T et al. *JAMA Network Open.*

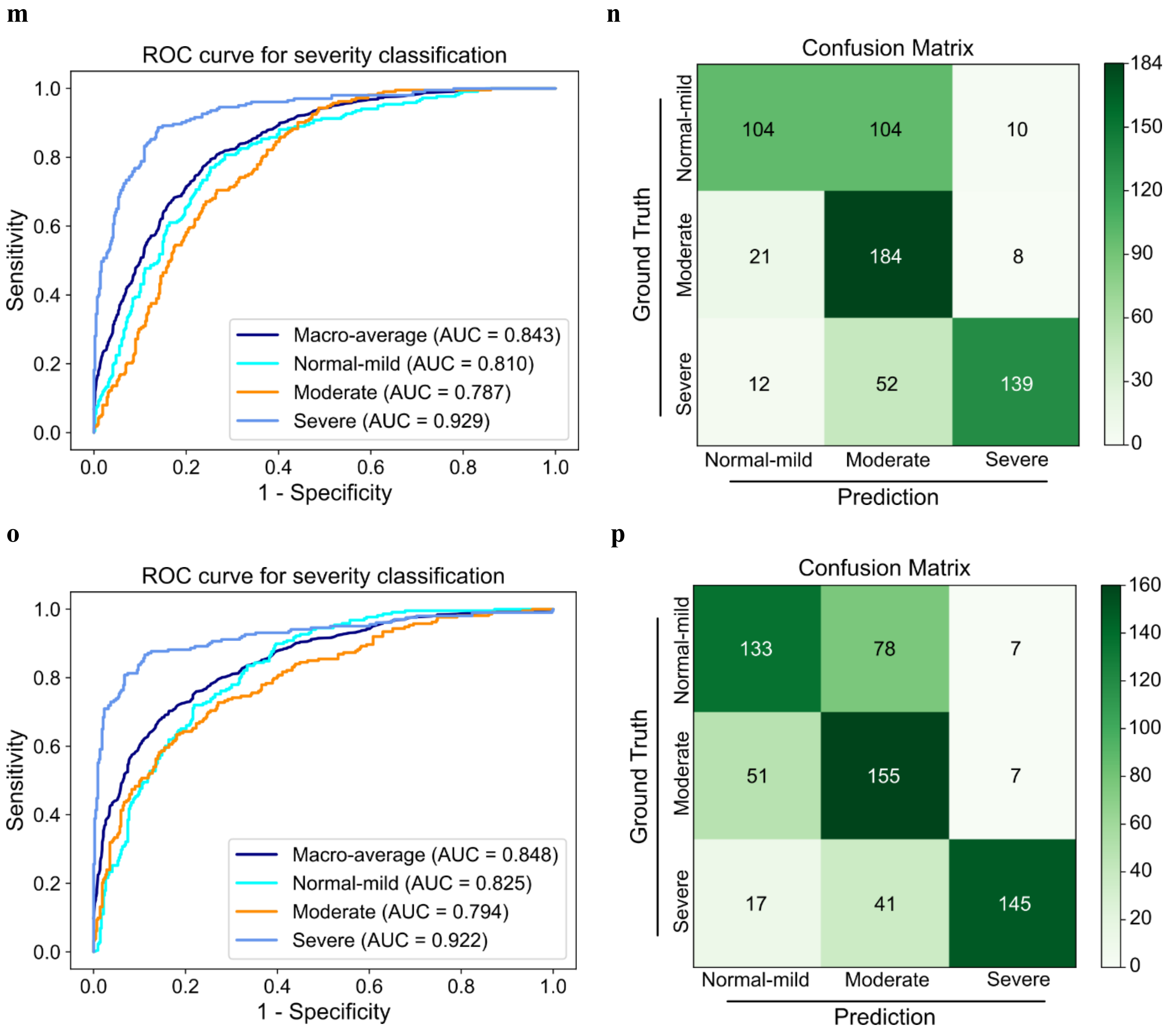

eFigure 4. ROC Curves and Confusion Matrices for Severity Classification in the In-House Validation Tests of Different Models

a-b, ROC curve and confusion matrix for *ScolioNets*. Compared with the other models, *ScolioNets* has the best performance; **c-d**, ROC curve and confusion matrix for *ResNet50 with attention*; **e-f**, ROC curve and confusion matrix for *DenseNet169 with attention;* **g-h**, ROC curve and confusion matrix for *ResNet50*; **i-j**, ROC curve and confusion matrix for *DenseNet169*; **k-l**, ROC curve and confusion matrix for *ResNeXt50*; **m-n**, ROC curve and confusion matrix for *Vgg16*; **o-p**, ROC curve and confusion matrix for *Inception-V4*.

© 2023 Zhang T et al. *JAMA Network Open.*

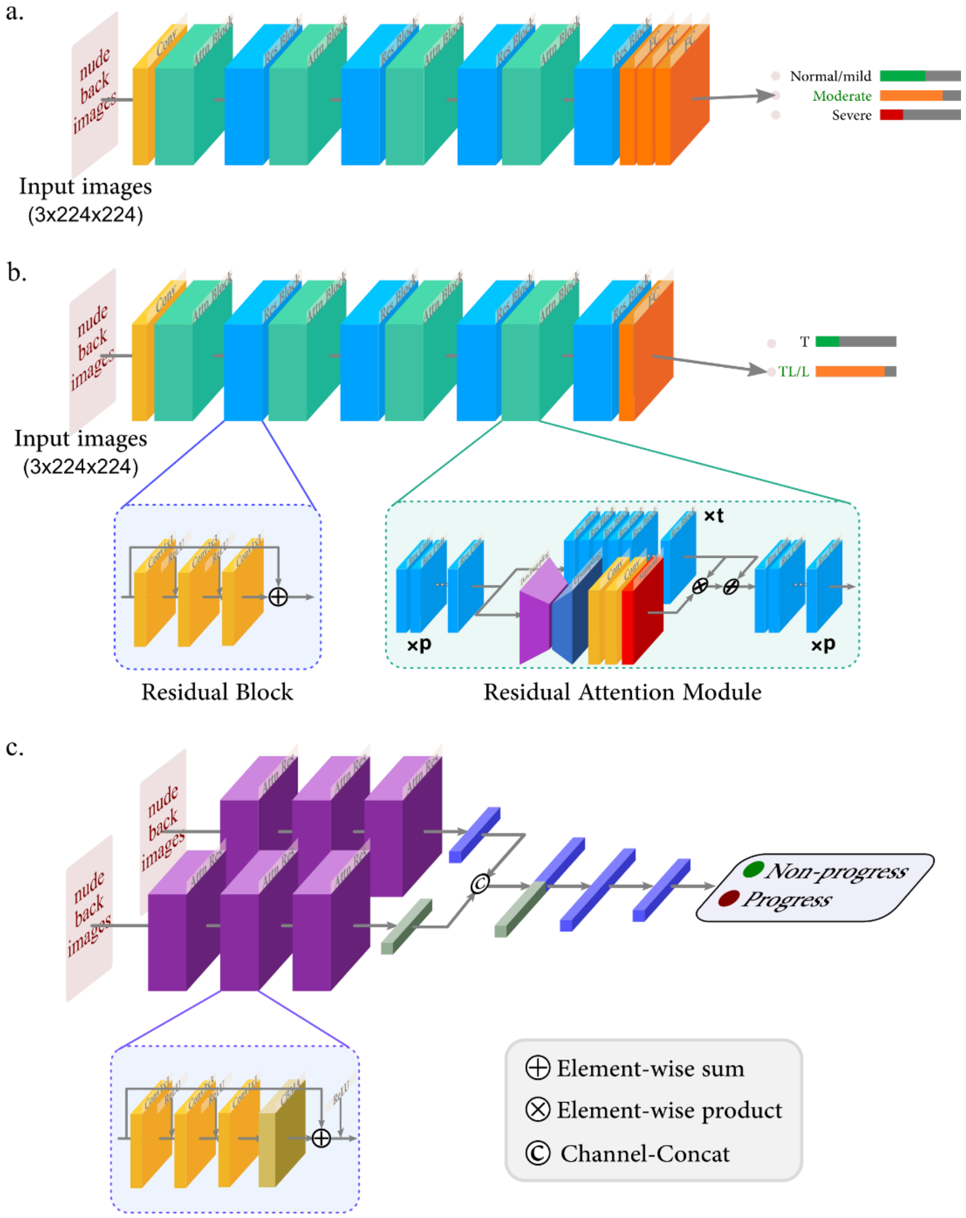

eFigure 5. The Detailed Architecture of ScolioNets for Severity Grading, Curve Type Classification, and Follow-Up Progression Prediction

severity grading (a), curve type classification (b) and FUs progression prediction (c). The input size is resized to 3×224×224 fora, b and c. To develop *ScolioNets*, Resnet50 with residual attention module is used as feature extractor. Residual blocks and attention blocks are alternately connected in series. Three parallel fully connected layers are used as classifiers at last. The residual block consists of several residual units, and the residual unit is just a sample, the number of convolutional layers and parameters vary for different blocks of units. One residual attention block consists of two branches called trunk branch and mask branch. The number of residual units in a residual attention block is controlled by the parameters '**p**' and '**t**'. Different attention stages have different depth of layers. The output of a is the severity of malformation and of b is the type of the curve. To deal with FUs progression prediction, two images are passed through two identical branches whose weights are not shared and then the two branches are concatenated and get the progression prediction through classifier.

© 2023 Zhang T et al. *JAMA Network Open.*

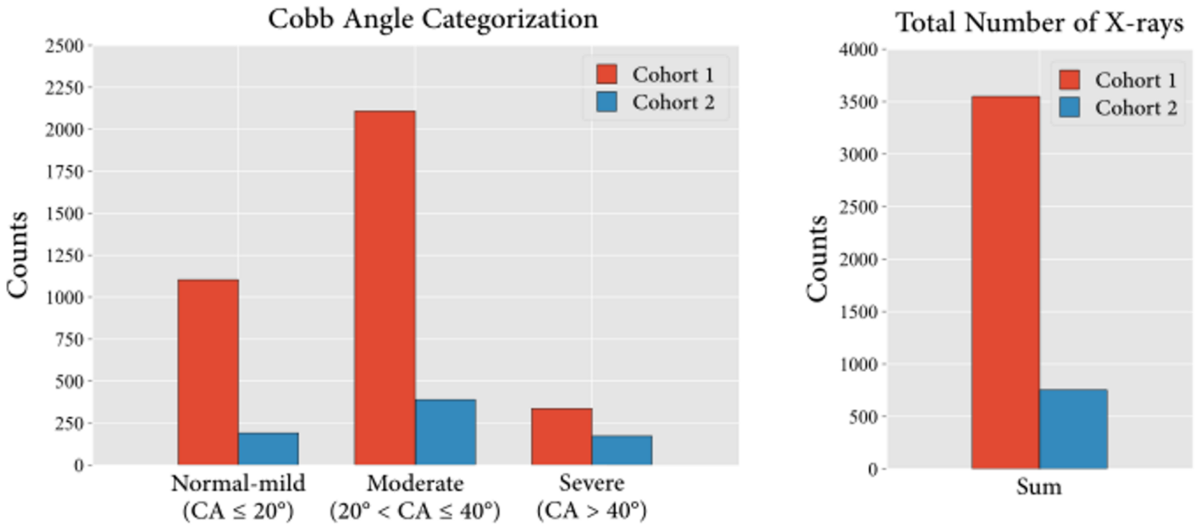

eFigure 6. Radiographs With Confirmed No or Mild, Moderate, and Severe Classifications in Cohorts 1 and 2

© 2023 Zhang T et al. *JAMA Network Open.*

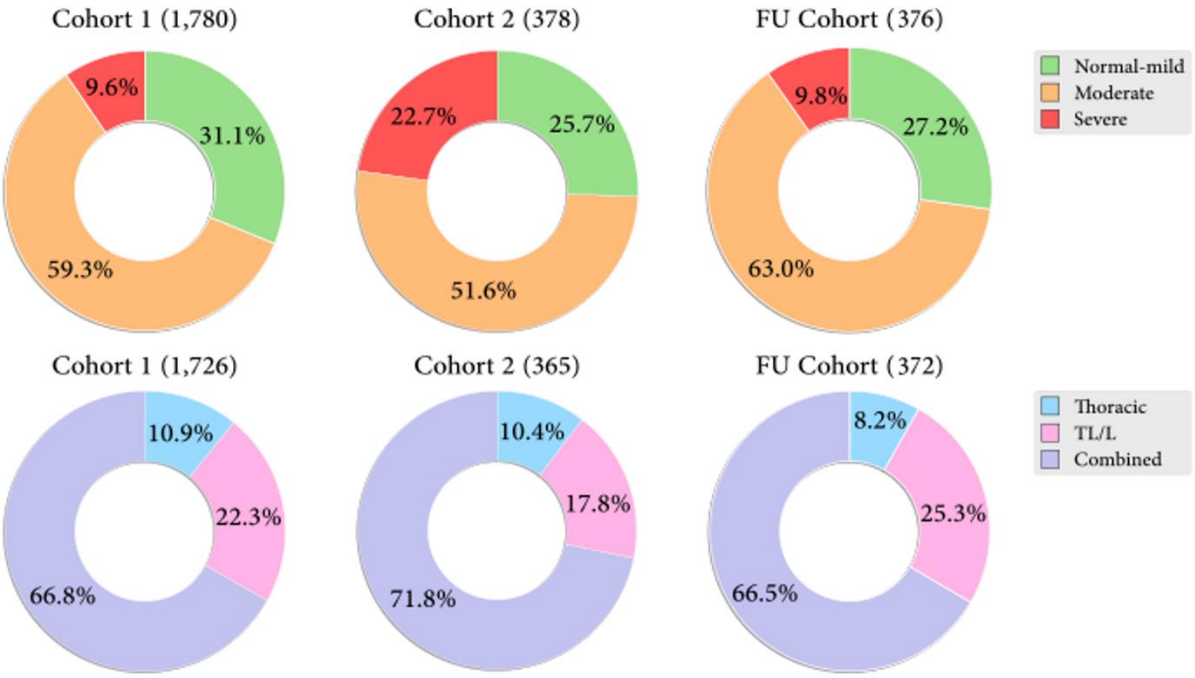

eFigure 7. Radiographs Taken for Each Severity Class and Curve Type

© 2023 Zhang T et al. *JAMA Network Open.*

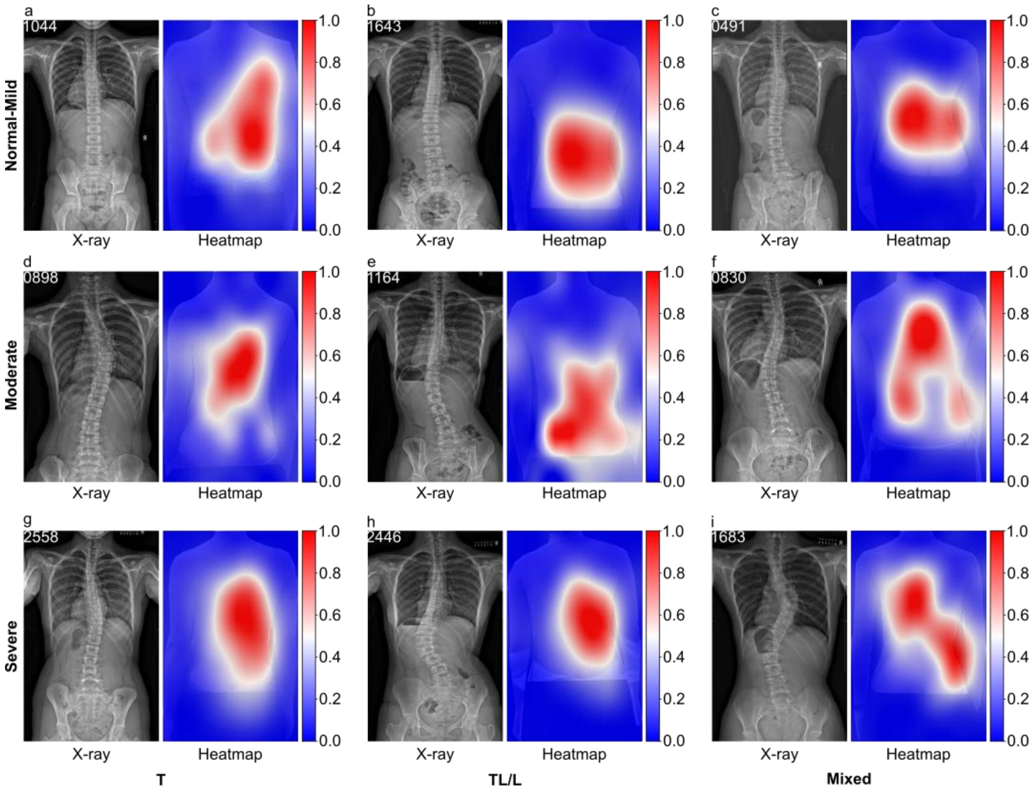

eFigure 8. Heatmaps of Areas Used to Support Classification Decisions

The interpretable heatmap reflects the areas in the image that are used to support classification decisions. In the heatmap, different colours are used to describe the degree of support for classification decisions in different regions in the image. From blue to red, the degree of support for classification decisions is increasing. Patient examples with different severities and curve types of spinal malformation were visualized based on the thermographic decision area.

© 2023 Zhang T et al. *JAMA Network Open.*

eTable 1. Comparative Results for In-House Validation Set

a, Comparison of the performance evaluation metrics between *ScolioNets* and other backbones on the in-house validation dataset requiring no interventions (<20°) or follow-up (FU) using single back photographs. It shows that *ScolioNets* has optimal and relatively balanced results with the highest AUC score. **b,** Comparison of the performance evaluation metrics between *ScolioNets* and other backbones on the in-house validation dataset in distinguishing severe curves (>40°; CS) using single back photographs. It shows that *ScolioNets* has optimal and relatively balanced results with the highest AUC score. The value of 95% CI in the square brackets is calculated by an open-source tool (https://www.medcalc.org/calc/diagnostic_test.php).

(a) Comparison of the performance evaluation metrics between *ScolioNets* and other backbones on the in-house validation dataset requiring no interventions (<20°) or follow-up (FU) using single back photographs

Backbone	Sensitivity (%)	NPV(%)	Specificity(%)	PPV(%)	ACC(%)	AUC
ScolioNets	82.57 (76.87-87.36)	89.05 (85.83-91.61)	74.28 (69.79-78.41)	62.72 (58.56-66.70)	77.13 (73.66-80.34)	0.869 (0.841-0.895)
**Res50+att.**	79.36 (73.37-84.53)	87.80 (84.67-90.37)	77.8 8 (73.58-81.78)	65.28 (60.80-69.51)	78.39 (74.98-81.54)	0.863 (0.834-0.889)
**Dense169+att.**	81.19 (75.36-86.15)	87.69 (84.30-90.43)	70.19 (65.54-74.55)	58.80 (54.86-62.63)	73.97 (70.37-77.35)	0.817 (0.785-0.846)
**Resnet50**	90.83 (86.19-94.31)	92.70 (89.25-95.10)	61.06 (56.19-65.77)	55.00 (51.83-58.13)	71.29 (67.60-74.79)	0.823 (0.791-0.852)
**Densenet169**	84.86 (79.40-89.35)	90.43 (87.30-92.86)	75.00 (70.55-79.09)	64.01 (59.88-67.95)	78.39 (74.98-81.54)	0.852 (0.822-0.879)
**ResNeXt50**	81.19 (75.36-86.15)	84.47 (80.28-87.91)	53.61 (48.68-58.48)	47.84 (44.82-50.87)	63.09 (59.20-66.86)	0.751 (0.716-0.784)
**VGG16**	80.28 (74.37-85.34)	87.39 (84.04-90.12)	71.63 (67.04-75.92)	59.73 (55.67-63.65)	74.61 (71.03-77.95)	0.810 (0.777-0.840)
**InceptionV4**	89.45 (84.59-93.19)	91.61 (88.03-94.18)	60.34 (55.46-65.07)	54.17 (51.00-57.30)	70.35 (66.62-73.88)	0.825 (0.793-0.854)

NPV = negative predicted value; PPV = positive predicted value; ACC = Accuracy; AUC = area under curves (AUCs) of the receiver operating characteristic curve.

(b) Comparison of the performance evaluation metrics between *ScolioNets* and other backbones on the in-house validation dataset in distinguishing severe curves (>40°; CS) using single back photographs

Backbone	Sensitivity(%)	NPV(%)	Specificity(%)	PPV(%)	ACC(%)	AUC
ScolioNets	84.73 (79.03-89.38)	93.00 (90.57-94.84)	95.59 (93.20-97.33)	90.05 (85.32-93.38)	92.11 (89.73-94.09)	0.958 (0.939-0.972)
**Res50+att.**	83.74 (77.93-88.54)	92.33 (89.79-94.27)	92.11 (89.15-94.48)	83.33 (78.27-87.41)	89.43 (86.77-91.72)	0.947 (0.927-0.963)
**Dense169+att.**	93.60 (89.30-96.55)	96.46 (94.14-97.88)	82.13 (78.19-85.64)	71.16 (66.77-75.19)	85.80 (82.84-88.43)	0.946 (0.926-0.963)
**Resnet50**	84.24 (78.48-88.96)	91.88 (89.14-83.97)	83.99 (80.18-87.33)	71.25 (66.45-75.62)	84.07 (80.98-86.83)	0.928 (0.905-0.947)
**Densenet169**	88.18 (82.92-92.28)	93.72 (91.09-95.61)	83.06 (79.18-86.48)	71.03 (66.42-75.25)	84.70 (81.66-87.41)	0.920 (0.896-0.940)
**ResNeXt50**	80.30 (74.15-85.53)	89.56 (86.61-91.91)	79.58 (75.46-83.29)	64.94 (60.30-69.31)	79.81 (76.47-82.87)	0.879 (0.851-0.903)
**VGG16**	88.18 (82.92-92.28)	93.92 (91.38-95.75)	86.08 (82.45-89.21)	74.90 (70.12-79.14)	86.75 (83.86-89.29)	0.929 (0.906-0.948)
**InceptionV4**	86.21 (80.69-90.63)	93.19 (90.64-95.08)	88.86 (85.51-91.67)	78.48 (73.52-82.72)	88.01 (85.23-90.44)	0.922 (0.898-0.942)

NPV = negative predicted value; PPV = positive predicted value; ACC = Accuracy; AUC = area under curves (AUCs) of the receiver operating characteristic curve.

© 2023 Zhang T et al. *JAMA Network Open.*

eTable 2. Significance Level Results

a, the ***p*** value of Wilcoxon signed-rank test between *ScolioNets* and other models on the in-house validation set **b,** the ***p*** value of Wilcoxon signed-rank test between *ScolioNets* and Surgeons on the independent testing set **c,** the ***p*** value of Wilcoxon signed-rank test between Senior Surgeon and Junior Surgeon on the independent testing set. The value p<0.0001 is considered statistically significant.

**(a) *p* values of Wilcoxon signed-rank test between *ScolioNets* and other models on the in-house validation set**		
Backbone	FU	CS
**Res50+att.**	0.0247	0.1015
**Dense169+att.**	0.2050	<0.0001
**Resnet50**	<0.0001	<0.0001
**Densenet169**	0.8608	<0.0001
**ResNeXt50**	<0.0001	<0.0001
**VGG16**	0.6242	<0.0001
**InceptionV4**	<0.0001	1.0421e-4

**(b) *p* values of Wilcoxon signed-rank test between *ScolioNets* and Surgeons on the independent testing set**					
Surgeon	FU	CS	T	TL/L	Mixed
**Senior Surgeon**	0.0971	0.0195	<0.0001	0.0053	<0.0001
**Junior Surgeon**	0.0009	0.0033	<0.0001	<0.0001	<0.0001

**(c) *p* values of Wilcoxon signed-rank test between Senior Surgeon and Junior Surgeon on the independent testing set**	
FU	0.0874
CS	0.4328
T	<0.0001
TL/L	<0.0001
Mixed	<0.0001

FU: follow-up; CS: considering surgeries; T: thoracic; TL/L: thoracolumbar/lumbar

© 2023 Zhang T et al. *JAMA Network Open.*

eTable 3. Comparison of the Performance Evaluation Metrics Between ScolioNets and Surgeons on the Prospective Dataset in Distinguishing Severe Curves for Surgical Intervention Consideration Using Single Back Photographs

Method	Sen(%)	NPV(%)	Spe(%)	PPV(%)	ACC(%)	AUC
ScolioNets	82.56 (72.87-89.90)	90.00 (84.95-93.48)	78.49 (71.59-84.38)	65.74 (58.67-72.18)	79.84 (74.42-84.57)	0.902 (0.859-0.936)
**Senior Surgeon**	20.93	70.43	94.19	64.29	69.77	——
**Junior Surgeon**	19.76	70.51	95.93	70.83	70.54	——

Note: Sen=sensitivity; NPV=negative predictive value; Spe=specificity; PPV=Positive predictive value; ACC=accuracy; AUC=area under curve. The number inside the brackets denotes the 95% confidence intervals.

© 2023 Zhang T et al. *JAMA Network Open.*

eTable 4. Comparison of the Performance Evaluation Metrics Between ScolioNets and Surgeons on the Prospective Dataset in Distinguishing Curve Types Using Single Back Photographs

Method		Sen(%)	NPV(%)	Spe(%)	PPV(%)	ACC(%)	AUC
ScolioNets	T	82.31 (78.51-85.70)	61.97 (56.45-67.20)	54.10 (47.62-60.47)	77.10 (74.48-79.52)	72.51 (69.04-75.78)	0.777 (0.745-0.808)
	TL/L	81.18 (77.29-84.66)	62.11 (56.85-67.11)	57.55 (51.10-63.83)	78.11 (75.39-80.60)	72.93 (69.48-76.19)	0.760 (0.727-0.791)
	Mixed	87.32 (82.10, 91.48)	92.52 (89.64, 94.65)	68.30 (63.97, 72.41)	54.55 (51.06, 57.98)	74.07 (70.66, 77.28)	0.860 (0.834-0.887)
**Senior Surgeon**	T	76.64	58.04	60.66	78.52	71.08	——
	TL/L	75.49	55.56	57.14	76.67	69.09	——
	Mixed	41.31	75.35	78.12	45.13	66.95	——
**Junior Surgeon**	T	100.00	N/A	0	65.24	65.24	——
	TL/L	100.00	N/A	0	65.10	65.10	——
	Mixed	100.00	N/A	0	30.34	30.34	——

© 2023 Zhang T et al. *JAMA Network Open.*

eReferences.

1. Negrini S, Donzelli S, Aulisa AG, et al. 2016 SOSORT guidelines: orthopaedic and rehabilitation treatment of idiopathic scoliosis during growth. *Scoliosis Spinal Disord.* 2018;13:3.
2. Chlap P, Min H, Vandenberg N, Dowling J, Holloway L, Haworth A. A review of medical image data augmentation techniques for deep learning applications. *J Med Imaging Radiat Oncol.* 2021;65(5):545-563.
3. Shorten C, Khoshgoftaar TM. A survey on Image Data Augmentation for Deep Learning. *J Big Data-Ger.* 2019;6(1).
4. Zhang K, Liang J, Van Gool L, Timofte R. Designing a practical degradation model for deep blind image super-resolution. Paper presented at: International Conference on Computer Vision2021; Virtual.
5. He K, Zhang X, Ren S, Sun J. Deep Residual Learning for Image Recognition. Paper presented at: The IEEE Conference on Computer Vision and Pattern Recognition (CVPR)2016; Las Vegas.
6. Huang G, Liu Z, Van Der M, Lilian QW. Densely connected convolutional networks. Paper presented at: Proceedings of the IEEE conference on computer vision and pattern recognition (CVPR)2017.
7. Simonyan K, Zisserman A. Very deep convolutional networks for large-scale image recognition. In: arXiv:1409.1556; 2014.
8. Szegedy C, Loffe S, Vanhoucke V, Alemi AA. Inception-v4, inception-resnet and the impact of residual connections on learning. Paper presented at: Thirty-First AAAI Conference on Artificial Intelligence (AAAI-17)2017.
9. Xie S, Girshick R, Dollár P, Tu Z, He K. Aggregated residual transformations for deep neural networks. Paper presented at: IEEE conference on computer vision and pattern recognition2017.
10. Niu ZY, Zhong GQ, Yu H. A review on the attention mechanism of deep learning. *Neurocomputing.* 2021;452:48-62.
11. Fukui H, Hirakawa T, Yamashita T, Fujiyoshi H. Attention Branch Network: Learning of Attention Mechanism for Visual Explanation. Paper presented at: IEEE/CVF Conference on Computer Vision and Pattern Recognition2019.
12. Xie ZL, Chen JL, Feng Y, Zhang KY, Zhou ZT. End to end multi-task learning with attention for multi-objective fault diagnosis under small sample. *J Manuf Syst.* 2022;62:301-316.
13. Liu S, Johns E, Davison AJ. End-to-end multi-task learning with attention. Paper presented at: IEEE/CVF conference on computer vision and pattern recognition2019.
14. Zhao S, Liu T, Zhao S, Wang F. A neural multi-task learning framework to jointly model medical named entity recognition and normalization. Paper presented at: AAAI Conference on Artificial Intelligence2019.
15. Hu J, Shen L, Sun G. Squeeze-and-excitation networks. Paper presented at: IEEE conference on computer vision and pattern recognition2018.
16. Wang F, Jiang M, Qian C, et al. Residual attention network for image classification. Paper presented at: IEEE conference on computer vision and pattern recognition2017.

© 2023 Zhang T et al. *JAMA Network Open.*
