# Supplementary material for: Deep Learning Model to Classify and Monitor Idiopathic Scoliosis in Adolescents Using a Single Smartphone Photograph
Source: JAMA Netw Open. 2023 Aug 23;6(8):e2330617. doi: 10.1001/jamanetworkopen.2023.30617 (PMC10448299; doi:10.1001/jamanetworkopen.2023.30617)
Supplement: Supplement 2. — Data Sharing Statement [file jamanetwopen-e2330617-s002.pdf]

Data Sharing Statement

Zhang. Deep Learning Model to Classify and Monitor Idiopathic Scoliosis in Adolescents Using a Single Smartphone Photograph. *JAMA Netw Open*. Published August 23, 2023. doi:10.1001/jamanetworkopen.2023.30617

Data
Data available: Yes
Data types: Other (please specify)
Additional Information: De-identified images.
How to access data: Please contact cheungjp@hku.hk for the 378 images.
When available: With publication

Supporting Documents
Document types: None

Additional Information
Who can access the data: Researchers whose proposed use of the data has been approved
Types of analyses: Research purpose.
Mechanisms of data availability: With a signed data access agreement
